# Identification of Anticancer Targets in Ovarian Cancer Using Genomic Drug Sensitivity Data

**DOI:** 10.3390/ijms26199530

**Published:** 2025-09-29

**Authors:** Yebin Son, Jae Yong Ryu

**Affiliations:** 1 Department of Biotechnology, Duksung Women’s University, 33 Samyang-Ro 144-Gil, Dobong-gu, Seoul 01369, Republic of Korea; term410@duksung.ac.kr; 2 Department of Biomedical Systems Engineering, Soongsil University, 369 Sangdo-ro, Dongjak-gu, Seoul 06978, Republic of Korea

**Keywords:** ovarian cancer, drug sensitivity, PARP inhibitors, biomarker, gene dependency score, target discovery

## Abstract

PARP inhibitors exploit synthetic lethality in *BRCA1/2*-mutated ovarian cancers but are limited by emerging therapeutic resistance. Therefore, novel biomarkers predicting PARP inhibitor response are urgently needed. In this study, we performed integrative analysis using drug sensitivity, patient survival, gene dependency, and expression data to identify biomarkers associated with PARP inhibitor response in ovarian cancer. Mutations in *BRCA1*, *MLL2*, *NF1*, and *SMARCA4* were associated with increased sensitivity to PARP inhibitors, suggesting potential synthetic lethality with PARP1. In contrast, *SMAD4* mutations were linked to PARP inhibitor resistance, and low *SMAD4* expression was associated with poor overall survival in patients with ovarian cancer. Further gene dependency score (GDS)-based screening revealed 51 candidate genes potentially involved in *SMAD4*-mediated resistance. Functional enrichment revealed associations with stress response, tumor-associated signaling pathways, and additional processes. Subsequent correlation and survival analyses nominated *ACACA*, *PRPF4B*, and *TUBD1* as potential therapeutic targets. Notably, low *ACACA* expression in patients with low *SMAD4* expression was associated with improved survival, indicating its relevance in overcoming PARP inhibitor resistance. This study contributes to predicting clinical outcomes in ovarian cancer and developing personalized treatment strategies.

## 1. Introduction

Ovarian cancer is the seventh most common malignancy affecting women worldwide, with approximately 239,000 new cases and 152,000 deaths annually [1]. Although its incidence and mortality rates have gradually declined over time, ovarian cancer remains the most lethal gynecologic malignancy [2]. This is primarily because ovaries are anatomically located deep within the abdominal cavity; therefore, early symptoms are often nonspecific or unnoticed, leading to delayed diagnosis at advanced stages. The five-year relative survival rate, which is widely used in cancer diagnostics to indicate the probability of patient survival within five years of diagnosis, is 70–90% in the early stages of ovarian cancer [3]. However, this rate precipitously declines to approximately 30% in the advanced stages, contributing to an overall poor prognosis [4].

Furthermore, high-grade serous ovarian carcinoma, which has the most unfavorable prognosis among ovarian cancer subtypes, is the most commonly diagnosed subtype [4]. The etiology of this subtype is primarily attributed to genetic deficiencies, specifically homologous recombination deficiency (HRD) [5]. HRD is a state in which the homologous recombination repair pathway is dysfunctional. Homologous recombination is an essential cellular mechanism for repairing double-strand DNA breaks [6]. However, mutations in genes such as *BRCA1/2* can impede this repair process, leading to HRD. A promising therapeutic strategy targeting this mechanism involves the use of poly (ADP-ribose) polymerase (PARP) inhibitors, which block PARP1-mediated single-strand DNA break repair, leading to the accumulation of double-strand breaks, which require the BRCA1/2 pathway for repair [7]. In cancer cells with *BRCA1/2* mutations, this creates a synthetic lethal interaction where PARP1 inhibition selectively induces cancer cell death while sparing normal cells [8,9].

Synthetic lethality is a process where the simultaneous inactivation of two genes results in cell death, whereas the loss of either gene alone does not affect cell viability [10]. In 1997, Hartwell et al. first proposed the application of synthetic lethality in anticancer drug discovery [11]. PARP inhibitors exemplify the inaugural clinical implementation of synthetic lethality principles for targeted cancer treatment [12]. Olaparib, the first-in-class PARP inhibitor, was initially approved by the FDA in 2014 for treating ovarian cancer with a specific genetic mutation, known as “BRCA-mutated” ovarian cancer. Since then, its therapeutic scope has expanded significantly to encompass other cancer types, including breast and pancreatic cancers [13,14]. Anticancer agents based on synthetic lethality can achieve lethal effects selectively on tumor cells rather than those on normal cells, eventually reducing side effects and enhancing therapeutic efficacy compared to those of conventional chemotherapeutics [6].

Significant advances have been achieved in ovarian cancer treatment through the introduction of novel targeted therapies, such as bevacizumab [15], thus remarkably improving patient survival. However, challenges such as post-treatment recurrence and drug resistance remain unresolved, thereby limiting improvements in long-term survival rates. In particular, resistance to PARP inhibitors is frequently observed clinically and potentially occurs through various mechanisms, including the activation of compensatory pathways and restoration of *BRCA* function [16].

Synthetic rescue, an emerging concept of drug resistance, is defined as a genetic interaction where a cell modifies one gene to evade the deleterious effects of another inactivated gene [17], resulting in resistance to anticancer therapies [18]. Synthetic rescue interactions are classified into downregulated and downregulated (DD), where the inactivation of two genes promotes cell survival and downregulated and upregulated (DU), where the inactivation of one gene is compensated by the overexpression of another [17]. Cancer cells acquire drug resistance by activating alternative pathways that compensate for the functional loss of target genes. Consequently, the identification and inhibition of genes involved in synthetic rescue relationships that confer resistance to targeted anticancer agents can potentially overcome resistance and enhance therapeutic efficacy.

In this study, we aimed to perform an integrative analysis of genomic drug sensitivity data and genetic functional datasets to identify the genes associated with sensitivity (*BRCA1*, *MLL2*, *NF1*, and *SMARCA4*) and resistance, specifically *SMAD4*, whose reduced expression emerged as a prognostic marker, to PARP inhibitors in ovarian cancer. We also predicted potential synthetic lethality and synthetic rescue relationships between these genes and *PARP1*. Furthermore, through expression correlation and survival analysis, we proposed a novel therapeutic target gene (i.e., *ACACA*) and suggested a combinatorial strategy in which *ACACA* inhibition may overcome *SMAD4*-low–mediated resistance, thereby contributing to improved prognosis and the establishment of personalized treatment strategies for patients with ovarian cancer.

## 2. Results and Discussion

### 2.1. Identification of Sensitivity and Resistance Biomarkers to PARP Inhibitors

**Figure 1:**
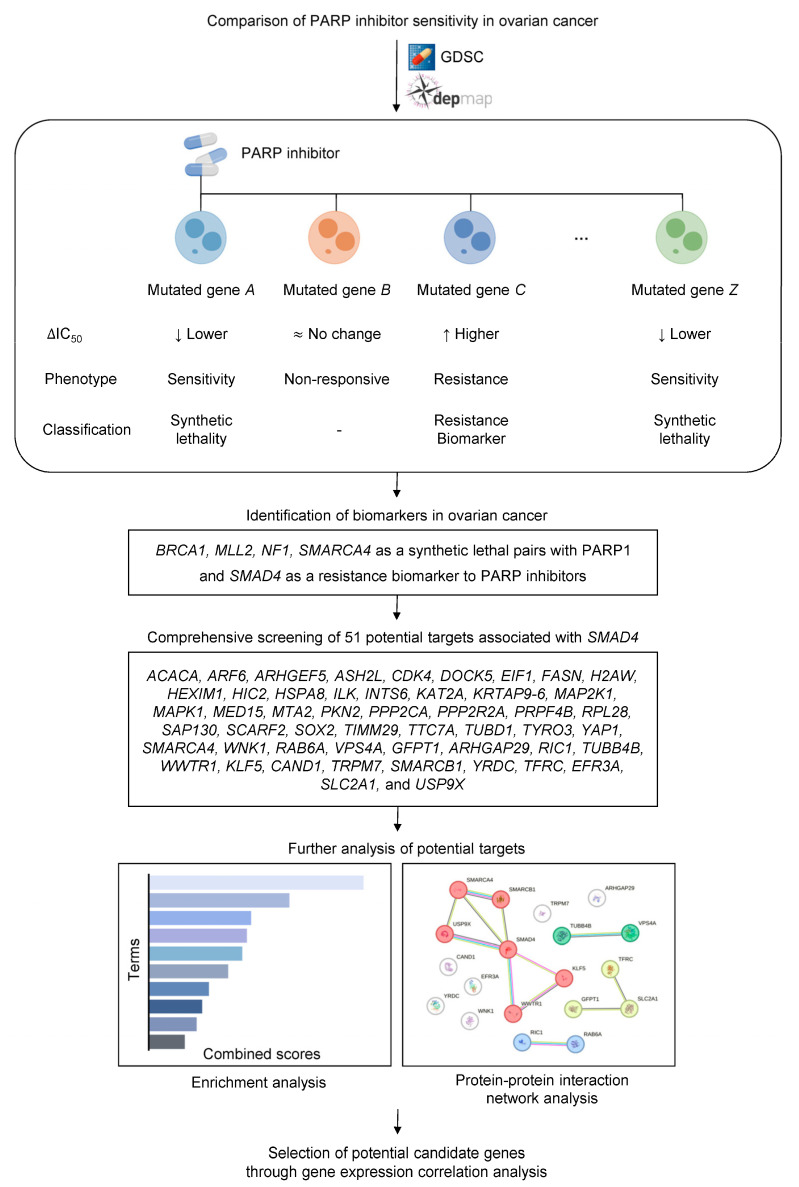
Overall scheme of the study. The workflow illustrates the data acquisition approach using the Genomics of Drug Sensitivity in Cancer (GDSC) database and DepMap portal to analyze drug sensitivity patterns. Changes in IC_50_ values (∆IC_50_) between wild-type and mutated genes were categorized into the following three phenotypes: lower IC_50_ indicating sensitivity, no change indicating non-responsiveness, and higher IC_50_ indicating resistance. Following drug sensitivity analysis, enrichment and protein–protein interaction network analyses were performed to identify additional potential targets. In the protein–protein interaction network analysis, the circles represent proteins.

**Figure 2:**
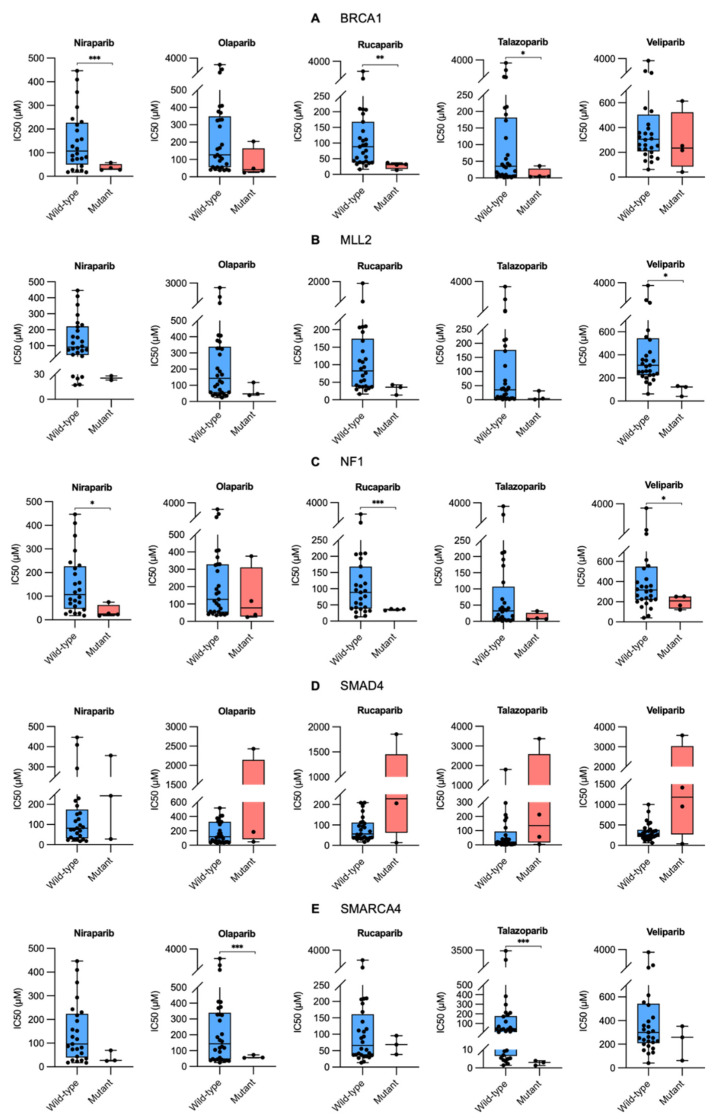
Drug sensitivity analysis of PARP inhibitors in relation to gene mutations in ovarian cancer cell lines. Box plots comparing the IC50 values of five PARP inhibitors (Niraparib, Olaparib, Rucaparib, Talazoparib, and Veliparib) between wild-type (blue boxes) and mutant (red boxes) ovarian cancer cell lines for *BRCA1*, *MLL2*, *NF1*, *SMAD4*, and *SMARCA4*. The y-axis represents the IC_50_ values (µM) for each drug, indicating the concentration required to inhibit cell growth by 50%. * *p* < 0.05, ** *p* < 0.01, *** *p* < 0.001.

In this study, we analyzed the Genomics of Drug Sensitivity in Cancer (GDSC) and Cancer Dependency Map (DepMap) databases to identify biomarkers for PARP inhibitors in ovarian cancer (Figure 1). The IC_50_ values for the five PARP inhibitors were then calculated and compared between the groups (Figure 2 and Table 1).

Based on *BRCA1* mutation status, the IC_50_ values of all the five PARP inhibitors in the mutant group were significantly lower than those in the wild-type group, with notable differences observed for talazoparib and veliparib (Figure S1). Similarly, IC_50_ values for all the drugs based on the *MLL2* mutation status were significantly lower in the mutant group than in the wild-type group, with significantly low values for olaparib, talazoparib, and veliparib. The IC_50_ values for all the drugs based on the *NF1* mutation status also decreased remarkably in the mutant group, with significantly low values for talazoparib and veliparib. Similarly, *SMARCA4* mutations resulted in lower IC_50_ values than those in the wild-type group for all the drugs, with significant reductions observed for talazoparib and veliparib. In contrast to other candidate genes, *SMAD4* mutation status demonstrated higher IC_50_ values in the mutant group than those in the wild-type group for all the drugs, with significant increases noted for olaparib, talazoparib, and veliparib.

Changes in the IC_50_ values based on gene mutation status suggest the functional involvement of these genes in regulating sensitivity to PARP inhibitors [19,20,21]. Specifically, our drug sensitivity analysis revealed that ovarian cancer cell lines with mutations in *BRCA1*, *MLL2*, *NF1*, and *SMARCA4* exhibited significantly decreased IC_50_ values for PARP inhibitors, whereas *SMAD4* mutant cell lines showed markedly increased IC_50_ values. Overall, mutations in *BRCA1*, *MLL2*, *NF1*, and *SMARCA4* significantly enhance sensitivity to PARP inhibitors in ovarian cancer cell lines, whereas *SMAD4* mutations induce resistance. These findings suggest the potential interaction mechanisms between each gene and PARP inhibitors, indicating that *SMAD4* may serve as a novel biomarker mediating resistance to PARP inhibitors through synthetic rescue relationships, whereas *BRCA1*, *MLL2*, *NF1*, and *SMARCA4* may exhibit synthetic lethal relationships with PARP1.

Our results suggest that, in addition to the well-known *BRCA1*, PARP1 may also show synthetic lethal relationships with *MLL2*, *NF1*, and *SMARCA4* [22,23]. These findings are supported by predictions with high combined scores from the SynLethDB database (https://www.synlethdb.com/, accessed on 15 December 2023), reinforcing the reliability of our research results [24]. In particular, the clinical applicability of *BRCA1* based on its synthetic lethal relationship with PARP1 is well-established [9]. *MLL2*, *NF1*, and *SMARCA4* may also affect cell survival through functional or genetic interactions with PARP1 [25,26]. Moreover, based on previous studies suggesting that the synthetic lethal relationships demonstrated in specific cancer types may be effective in other cancers with similar mechanisms [27,28], the synthetic lethal relationship between *MLL2* and PARP1 proposed in our study may exhibit similar effects of increased PARP inhibitor sensitivity, not only in ovarian cancer, but also in other cancer types with high *MLL2* mutation frequencies [29]. The same drug sensitivity analysis based on the cBioPortal database (https://www.cbioportal.org/) yielded similar results for bladder cancer, which had the highest frequency of *MLL2* mutations (Figure S2). This important finding contributes to the expansion of the application range of synthetic lethality-based targeted therapeutic strategies across different cancer types.

Conversely, the consistent increase in the IC_50_ values of PARP inhibitors owing to *SMAD4* mutations implies a potential synthetic rescue association between *SMAD4* and PARP1. The DD-type synthetic rescue relationship shows a pattern similar to that of the increased drug resistance with *SMAD4* mutations in our study. Therefore, this could be interpreted as a potential DD-type synthetic rescue relationship, which explains the mechanisms of resistance to PARP inhibitors, providing important evidence for predicting therapeutic responses in ovarian cancer and for establishing targeted therapeutic strategies to overcome resistance.

Although these observations were derived from in silico analyses, they provide a rationale for subsequent functional investigations to elucidate the underlying synthetic lethal mechanisms. Nonetheless, the study is limited by statistical power, as the number of SMAD4-mutant cell lines analyzed was substantially smaller than that of wild-type counterparts. Accordingly, the findings should be regarded as exploratory and interpreted with caution.

Analysis of drug sensitivity across 30 cancer types revealed that only four cancer types—esophageal carcinoma, head and neck squamous cell carcinoma, ovarian serous cystadenocarcinoma, and pancreatic adenocarcinoma—had available *SMAD4* mutation data; among these, only ovarian cancer showed statistically significant results (Figure S5). This finding may reflect the distinct molecular background of ovarian cancer, which is characterized by a markedly higher prevalence of homologous recombination deficiency than that observed in most other cancer types. Therefore, further research on the cancer-type specificity of *SMAD4*-mediated PARP inhibitor resistance mechanisms is necessary.

### 2.2. Survival Analysis of Candidate Genes in Ovarian Cancer

**Figure 3:**
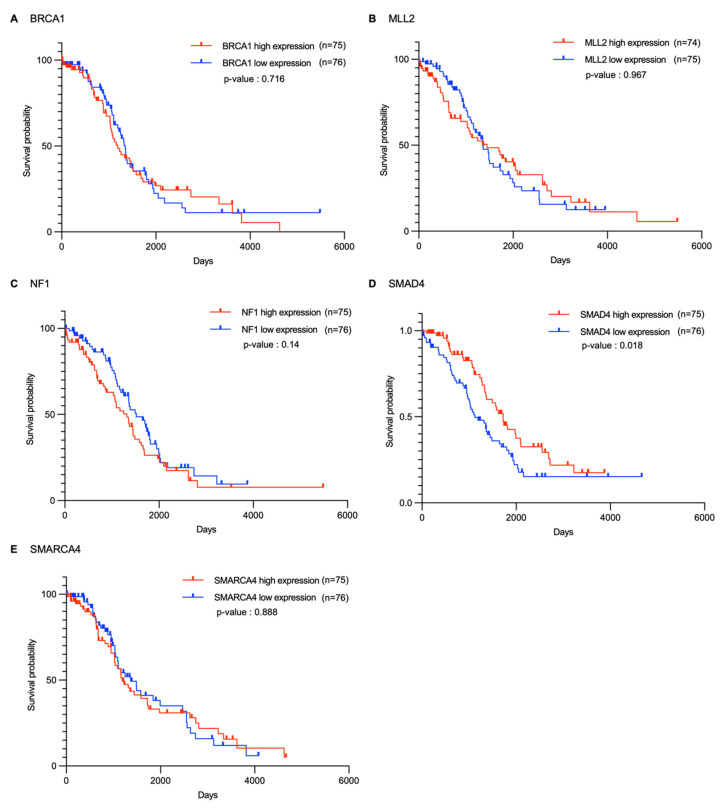
Kaplan–Meier survival curves comparing patients with ovarian cancer exhibiting high and low gene expression. Survival analysis of patients with ovarian cancer stratified by gene expression levels (highest and lowest 20%) of *BRCA1*, *MLL2*, *NF1*, *SMAD4*, and *SMARCA4*. The x-axis represents time in days, and the y-axis represents survival probability. Blue and red lines indicate patients with high and low gene expression, respectively. Statistical significance was determined using the log-rank test.

To support the drug sensitivity analysis results, we performed Kaplan–Meier survival analysis to evaluate the impact of *BRCA1*, *MLL2*, *NF1*, *SMAD4*, and *SMARCA4* expression levels on the survival of patients with ovarian cancer and to identify potential associations. The analysis was conducted based on ovarian cancer patient data obtained from The Cancer Genome Atlas (TCGA) database, comparing patient groups with the highest and lowest 20% expression levels for each gene (Figure 3). Survival analysis results were evaluated using the median survival time (MST), log-rank test statistic ($X^{2}$), *p*-value, hazard ratio (HR), and 95% confidence interval (CI) (Table S1).

The analysis revealed that only *SMAD4* showed a significant difference in survival rate (*p* = 0.0182). The MST of the high *SMAD4* expression group was 1720 days, which was significantly longer than that of the low *SMAD4* expression group, which was 1123 days; an HR of 0.6087 (95% CI: 0.4020–0.9218) was observed for the high expression group compared to that of the low expression group (Figure 3 and Table S1). Therefore, *SMAD4* expression levels are closely associated with patient survival, and lower expression levels correspond to a decreased survival probability. Considering the high IC_50_ values of PARP inhibitors in *SMAD4* mutant cell lines observed in drug sensitivity analyses, the survival analysis results support the possibility that *SMAD4* can function as a resistance biomarker for PARP inhibitors.

In contrast, no significant differences in survival rates based on expression levels were observed for *BRCA1*, *MLL2*, *NF1*, and *SMARCA4*. For *BRCA1*, the MSTs of the high and low expression groups were 1189 and 1348 days, respectively; the low expression group exhibited relatively long survival and HR = 1.079 (95% CI: 0.7153–1.627), indicating no significant difference in risk. Similarly, *SMARCA4* showed HR = 1.031 (95% CI: 0.6731–1.578), and *NF1* and *MLL2* showed *p* > 0.05, indicating no significant association between expression levels and survival rates.

Based on the survival analysis results, while mutations *BRCA1*, *MLL2*, *NF1*, and *SMARCA4* can be associated with drug responsiveness to PARP inhibitors, changes in expression levels alone may not directly affect long-term patient survival. Conversely, *SMAD4* showed consistent results in both drug resistance and decreased survival, suggesting its potential as a target biomarker for both prognostic prediction and treatment response. Notably, the low HR of approximately 0.6 in the survival analysis potentially indicates that the expression of a single gene can considerably impact clinical prognosis. Along with the drug sensitivity analysis findings, the survival analysis results confirm that *SMAD4* is a biomarker mediating resistance to PARP inhibitors in patients with ovarian cancer and may contribute to decreased patient survival rates. Furthermore, this analysis revealed that SMAD4 expression also serves as a prognostic marker.

### 2.3. Gene Dependency Score-Based Identification and Functional Analysis of SMAD4-Mediated Resistance

Based on the drug sensitivity and survival analyses results, SMAD4 was identified as a promising candidate biomarker for resistance to PARP inhibitors in ovarian cancer. Therefore, subsequent analyses were performed to identify the potential target genes for overcoming SMAD4-mediated resistance. Here, the term SMAD4-mediated resistance refers to the phenomenon where resistance to PARP inhibitors occurs when SMAD4 expression is reduced or deficient.

**Figure 4:**
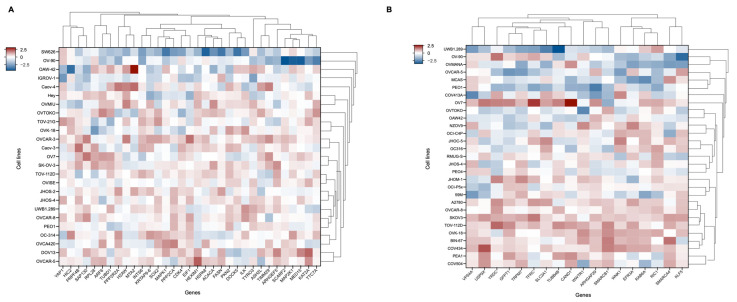
Heatmap analysis of gene dependency scores (GDS) in ovarian cancer cell lines based on *SMAD4* status. (**A**) Heatmap showing 33 genes with significantly different GDS values between *SMAD4* mutant and wild-type ovarian cancer cell lines. Cell lines are categorized as mutant (SW626, OV-90, IGROV-1) or wild-type along the y-axis, and target genes are displayed along the x-axis. (**B**) Heatmap depicting 18 genes with significantly different GDS values between ovarian cancer cell lines with high and low *SMAD4* expression. Cell lines are grouped by *SMAD4* expression levels along the y-axis, with low expression cell lines clustered at the top and high expression cell lines at the bottom, and target genes are displayed along the x-axis. Detailed cell line classifications are provided in Table 2. The color scale represents GDS values, with blue indicating lower scores (greater dependency) and red indicating higher scores (less dependency). Clustering was performed using Euclidean distance calculation and the average linkage method.

First, 33 ovarian cancer cell lines obtained from the GDSC and DepMap databases were divided into mutant and wild-type groups according to *SMAD4* mutation status and simultaneously grouped into high- and low-*SMAD4* expression groups, for analysis (Table 2). Subsequently, comparative analysis was conducted using Gene Dependency Score (GDS) data from the DepMap portal for each group [30,31]. Consequently, the following 33 candidate genes were identified based on *SMAD4* mutation status: *MAPK1*, *INTS6*, *TTC7A*, *MTA2*, *SOX2*, *MAP2K1*, *PPP2R2A*, *RPL28*, *TYRO3*, *KAT2A*, *ARHGEF5*, *DOCK5*, *HSPA8*, *HIC2*, *PKN2*, *SCARF2*, *MED15*, *ARF6*, *CDK4*, *TIMM29*, *HEXIM1*, *YAP1*, *ASH2L*, *TUBD1*, *H2AW*, *EIF1*, *PPP2CA*, *PRPF4B*, *ACACA*, *SAP130*, *KRTAP9-6*, *ILK*, and *FASN*. In addition, the following 18 candidate genes were selected based on expression levels: *SMARCA4*, *WNK1*, *RAB6A*, *VPS4A*, *GFPT1*, *ARHGAP29*, *RIC1*, *TUBB4B*, *WWTR1*, *KLF5*, *CAND1*, *TRPM7*, *SMARCB1*, *YRDC*, *TFRC*, *EFR3A*, *SLC2A1*, and *USP9X* (Table 3 and Table 4). In total, 51 genes were identified as potential targets based on *SMAD4* mutations or expression levels (Figure 4). These genes demonstrated an increased tendency for cancer cell survival in *SMAD4* mutant and low *SMAD4* expression cell groups compared to those in the control groups and can be proposed as candidates capable of effectively inhibiting cell growth. Furthermore, these genes can act as potential targets for overcoming therapeutic resistance in *SMAD4*-related ovarian cancer. Therefore, subsequent enrichment analyses were conducted, to elucidate the biological functions and pathways through which these genes are involved in cells and their functional association with *SMAD4* [32,33]. Enrichment analyses were performed using Enrichr (https://maayanlab.cloud/Enrichr/, accessed on 15 December 2023) databases and analytical tools, including Gene Ontology (GO) biological processes and pathway analyses [34].

**Figure 5:**
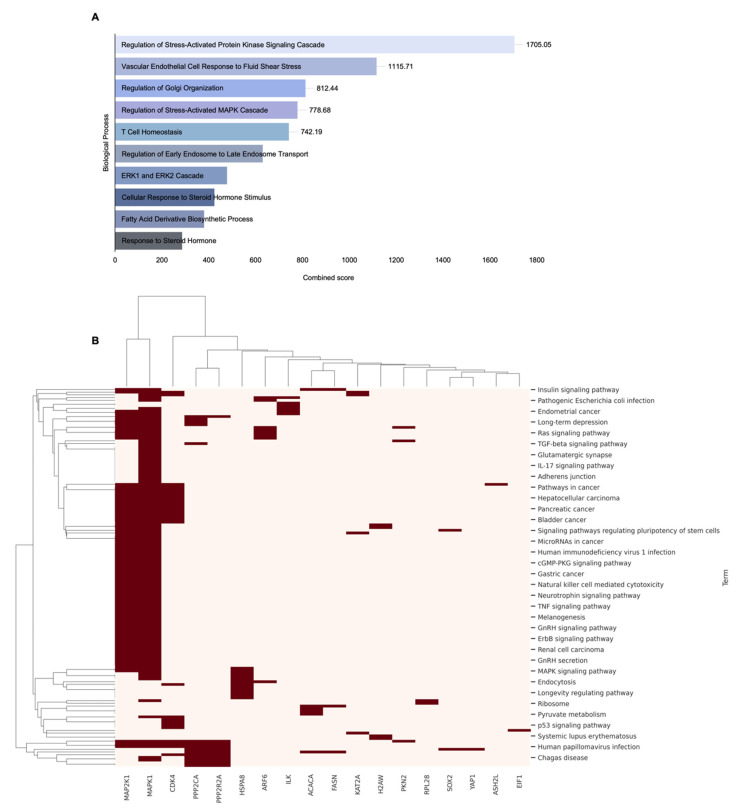
Enrichment analysis of 33 genes screened based on *SMAD4* mutation status. (**A**) Gene Ontology (GO) biological process analysis results, presented as a bar graph of the top 10 enriched processes sorted by the combined score. (**B**) Pathway analysis results are presented as a clustered heatmap with a dendrogram, showing genes significantly associated with multiple pathways. Only genes associated with two or more pathways and showing significant enrichment were included in the visualization. Clustering was performed using Euclidean distance calculation and the average linkage method between clusters. Red blocks indicate the association of genes (shown along the bottom axis) with the corresponding pathways.

GO biological process analysis was performed on the 33 potential target genes selected based on *SMAD4* mutation status (Table 3), to identify their major biological functions. On sorting the results based on the combined scores, these genes were found to be significantly enriched in various biological pathways, including metabolic pathways, stress responses, and intracellular transport (Figure 5A). In total, 468 enriched entries were identified, and notably, “Regulation of Stress-Activated Protein Kinase Signaling Cascade” emerged as a major pathway with the highest combined score of 1705.05, which included *MAPK1* and *MAP2K1*. Other highly ranked biological pathways included “Vascular Endothelial Cell Response to Fluid Shear Stress” (1115.71), “Regulation of Golgi Organization” (812.44), “Regulation of Stress-Activated MAPK Cascade” (778.68), and “T cell Homeostasis” (742.19), along with various pathways related to cellular structure maintenance, immune homeostasis, hormone response, and fatty acid biosynthesis. Subsequently, pathway analysis was performed on these genes, to identify their signaling and metabolic pathways. In total, 140 enriched entries were identified; specifically, among the top-ranked pathways based on combined scores, highly ranked pathways included “Bladder cancer” (528.78), “Endometrial cancer” (327.24), “Long-term depression” (312.19), “Sphingolipid signaling pathway” (239.58), “AMPK signaling pathway” (236.73), and “PI3K-Akt signaling pathway” (134.47). These pathways are known to contribute to cancer cell proliferation, survival, and metabolic adaptation [35]; the PI3K-Akt and AMPK signaling pathways are particularly recognized for their central roles in cancer cell viability and energy metabolism reprogramming [36,37,38]. Additionally, various tumor type-related pathways, such as Melanoma, Pancreatic cancer, Glioma, and Non-small cell lung cancer, as well as key signaling pathways, including Hippo signaling, TGF-β signaling, and mTOR signaling, were significantly enriched (Figure 5B). These results suggest that the candidate genes derived based on *SMAD4* mutations are involved in major functional pathways related to tumor signaling network reorganization, microenvironment regulation, and therapeutic resistance.

**Figure 6:**
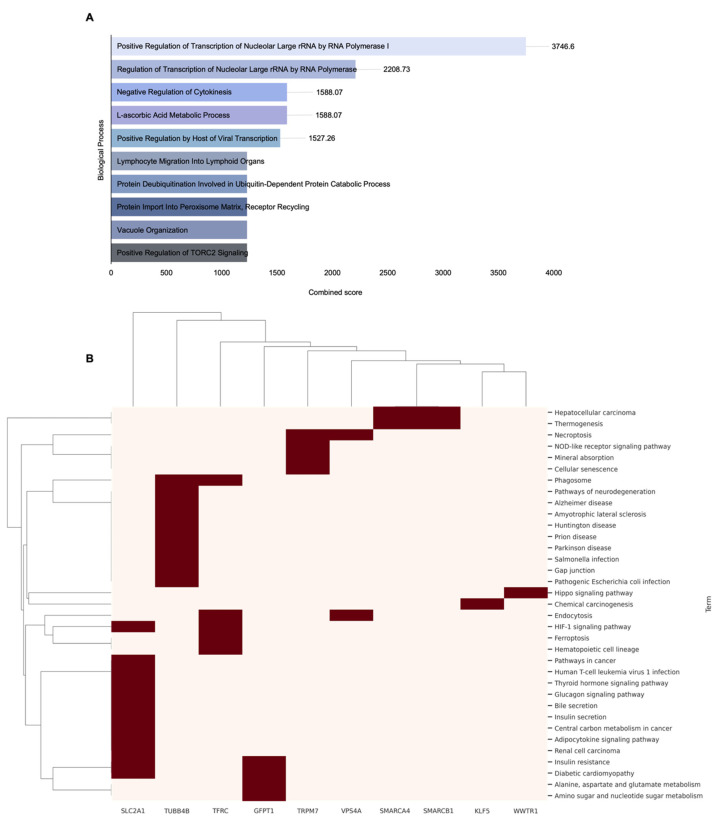
Enrichment analysis of 18 genes screened based on *SMAD4* expression levels. (**A**) GO biological process analysis results, presented as a bar graph of the top 10 enriched processes sorted by the combined score. (**B**) Pathway analysis results are presented as a clustered heatmap with a dendrogram, showing genes significantly associated with multiple pathways. Only genes associated with two or more pathways and those showing significant enrichment were included in the visualization. Clustering was performed using Euclidean distance calculation and the average linkage method between clusters. Red blocks indicate the association of genes (shown along the bottom axis) with the corresponding pathways.

Subsequently, GO biological process analysis was performed on the 18 potential target genes selected based on *SMAD4* expression levels (Table 4), to identify their major biological functions. When sorted according to combined scores, these genes showed significant associations, primarily with pathways related to transcriptional regulation, intracellular protein metabolism, immune cell migration, and metabolic response regulation (Figure 6A). In total, 369 enriched entries were identified, and the entry with the highest combined score was “Positive Regulation of Transcription of Nuclear Large rRNA by RNA polymerase I” with a score of 3746.6, including *SMARCA4* and *SMARCB1*. Other highly ranked pathways included “Regulation of Transcription of Nucleolar Large rRNA by RNA Polymerase” (2208.73), “Negative Regulation of Cytokinesis” (1588.07), “L-ascorbic Acid Metabolic Process” (1588.07), and “Lymphocyte Migration into Lymphoid Organs” (1227.64). Entries involving four or more genes included “Regulation of Transcription by RNA Polymerase II” and “Positive Regulation of Transcription by RNA Polymerase II,” suggesting that these genes are involved in transcriptional regulatory functions closely related to cell growth and proliferation. The inclusion of pathways related to rRNA transcriptional regulation and negative regulation of cytokinesis among the top entries suggests that these genes may significantly affect protein synthesis and cell cycle regulation. Subsequently, pathway analysis revealed that these genes were mainly involved in metabolic homeostasis maintenance, apoptosis regulation, and tumor-related signaling pathways (Figure 6B). In total, 35 enriched entries were identified, and in the specific results, the pathway with the highest combined score was “Insulin resistance” with a score of 128.40, which included *GFPT1* and *SLC2A1*. This pathway plays a crucial role in glucose metabolism and energy regulation and contributes to cancer cell adaptation in highly metabolic environments [39]. Additionally, the “HIF-1 signaling pathway” also ranked highly with a combined score of 126.77, including *TFRC* and *SLC2A1*. The HIF-1 signaling pathway is closely related to gene expression regulation in hypoxic environments and cancer cell survival strategies [40,41,42]. Other highly ranked pathways included “phagosomes” (79.57), “necroptosis” (74.63), “Hepatocellular carcinoma” (68.98), and “diabetic cardiomyopathy” (52.42), which included genes such as *TFRC*, *TRPM7*, *VPS4A*, *SMARCA4*, *SMARCB1*, and *VPS4A*. These pathways are associated with cancer cell apoptosis regulation, pathological metabolic changes, and tumor progression [43,44]. These results demonstrate that genes derived based on *SMAD4* expression levels are extensively involved in cellular transcription regulation, energy metabolism, stress responses, and survival-related signaling pathways. In summary, genes related to *SMAD4* expression, through their involvement in various cancer-related biological functions and pathways, can provide important biological clues for elucidating the molecular mechanisms underlying *SMAD4*-mediated drug resistance.

**Figure 7:**
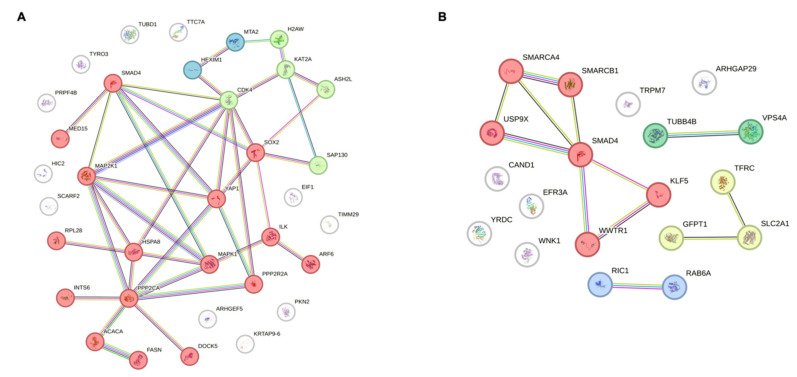
Protein–protein interaction (PPI) network analysis of potential target genes associated with *SMAD4*. Network visualization showing functional relationships between genes, where connecting lines indicate various types of associations, including co-expression, experimental/biochemical data, curated database entries, or co-mention in published literature. Genes showing direct interactions with *SMAD4* are highlighted in red, while other colors represent distinct functional clusters of genes associated with each other rather than directly with *SMAD4*. (**A**) PPI network of *SMAD4* and 33 genes screened based on *SMAD4* mutation status. (**B**) PPI network of *SMAD4* and 18 genes screened based on *SMAD4* expression levels. The analysis was performed using the STRING database. In the protein–protein interaction network analysis, the circles represent proteins.

To elucidate the functional association between *SMAD4* and the 51 potential target genes identified based on *SMAD4* mutation status and expression levels, protein–protein interaction (PPI) network analysis was performed using the STRING database and analytical tools (https://string-db.org/). Several genes exhibited moderate or stronger interactions, with *USP9X* (0.971), *YAP1* (0.943), *WWTR1* (0.875), *SOX2* (0.820), and *MAPK1* (0.816) recording combined scores of 0.8 or higher, indicating high potential for functional linkage with *SMAD4* (Figure 7). *USP9X*, which showed the highest combined score, is a protein-deubiquitinating enzyme and maintains protein stability by inhibiting the ubiquitination of *SMAD4* [45]. Indeed, in colorectal cancer cell models, *USP9X* expression inhibition decreases *SMAD4* protein levels, suggesting that *USP9X* is essential for *SMAD4*-mediated tumor suppression [46]. These results confirm that *USP9X* may also play an important role in regulating *SMAD4*-mediated resistance in ovarian cancer cells. Additionally, *YAP1* and *WWTR1*, which are core regulatory factors of the Hippo signaling pathway [47], showed high interaction scores of 0.943 and 0.875, respectively. These genes act as transcriptional cofactors that promote cancer cell growth and survival. Although *YAP1* showed weak co-expression with *SMAD4* in PPI analysis, it notably displayed a positive correlation with ovarian cancer. Furthermore, *MAPK1* (0.816) and *MAP2K1* (0.695) are major components of the *MAPK* signaling pathway that intersect with TGF-β signaling, potentially forming indirect functional links with *SMAD4*. *SOX2* (0.820), a transcription factor involved in maintaining pluripotency and regulating cell differentiation [48], suggests the possibility of a functional intersection with *SMAD4* in pathways related to targeted therapy resistance and cancer stem cell characteristics. In contrast, *SMARCB1* (0.514), *CDK4* (0.552), and *KLF5* (0.572) showed relatively low combined scores and their functional associations with *SMAD4* can be interpreted at a supplementary level. In particular, considering that the expression correlation pattern of *SMARCB1* with *SMAD4* in ovarian cancer appeared to be positive, the possibility of biological significance cannot be completely ruled out. Overall, these PPI analysis results indicate that multiple genes with direct or indirect functional links to *SMAD4* may be associated with PARP inhibitor resistance, and these genes hold potential as novel therapeutic targets for overcoming *SMAD4*-mediated drug resistance in ovarian cancer.

### 2.4. Correlation and Survival Analysis of SMAD4-Associated Genes

**Figure 8:**
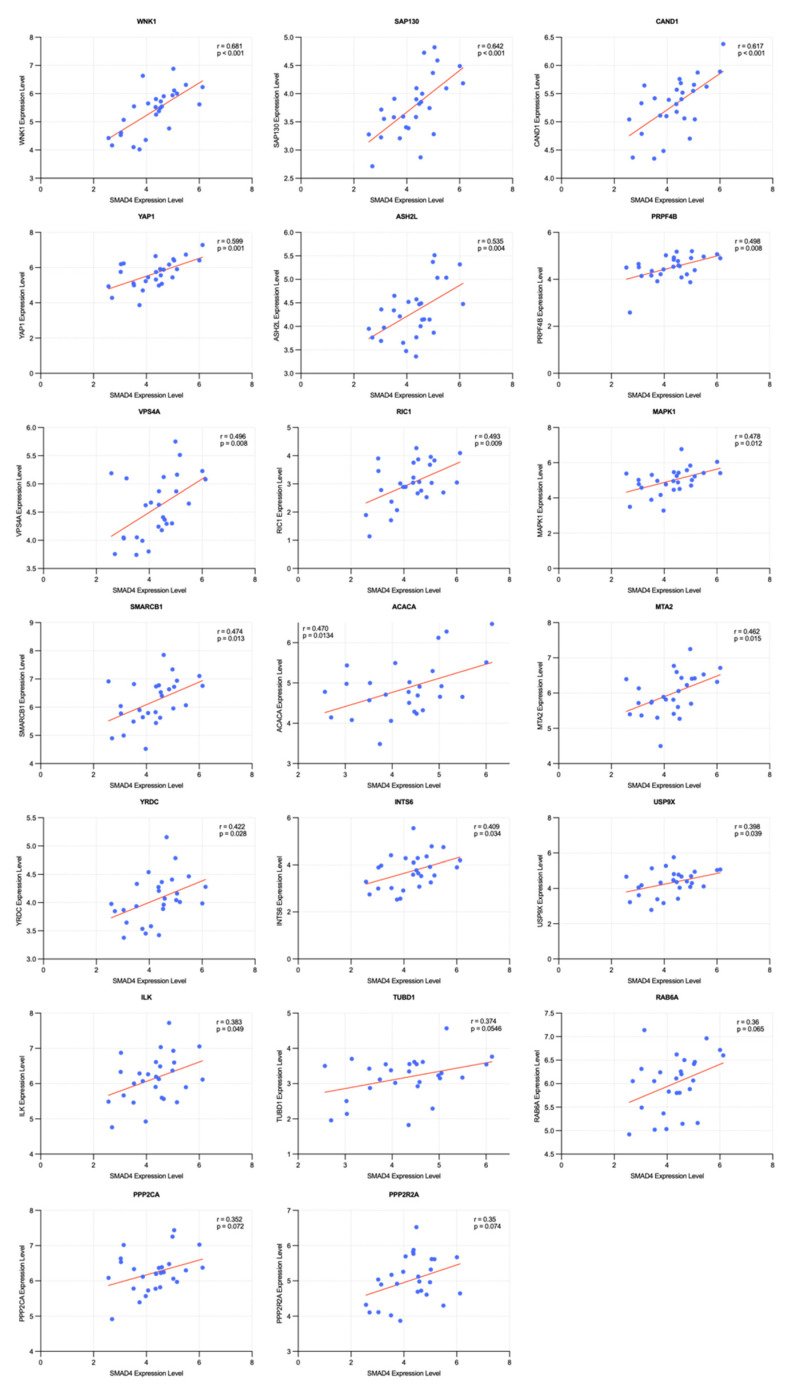
Correlation analysis of gene expression between *SMAD4* and potential target genes in ovarian cancer cell lines. Scatter plots showing the expression correlation between *SMAD4* (x-axis) and 20 potential target genes (y-axis) from 51 screened candidates. Genes are arranged in the order of decreasing Pearson correlation coefficient (*r*), with only those having absolute *r* values > 0.35 included. Each plot displays the correlation coefficient and *p*-value. Blue dots represent individual ovarian cancer cell lines, and red lines indicate the linear regression fit. Expression data were obtained from the DepMap portal, with raw data presented in Supplementary Table S2.

The 51 *SMAD4*-associated candidate genes identified in previous analyses were confirmed to be involved in various biological pathways and functional mechanisms. To verify their expression associations, Pearson’s correlation coefficient analysis was performed between each candidate gene and *SMAD4* in ovarian cancer cell lines (Figure S3). This analysis identified the following 20 genes at a threshold of |r|≥ 0.35, listed in descending order of correlation: *WNK1*, *SAP130*, *CAND1*, *YAP1*, *ASH2L*, *PRPF4B*, *VPS4A*, *RIC1*, *MAPK1*, *SMARCB1*, *ACACA*, *MTA2*, *YRDC*, *INTS6*, *USP9X*, *ILK*, *TUBD1*, *RAB6A*, *PPP2CA*, and *PPP2R2A*. All these genes positively correlated with *SMAD4* (Figure 8). *WNK1* (r = 0.681), *SAP130* (r = 0.642), and *CAND1* (r = 0.617) showed particularly high correlation coefficients, confirming their close association with SMAD4 expression regulation.

**Figure 9:**
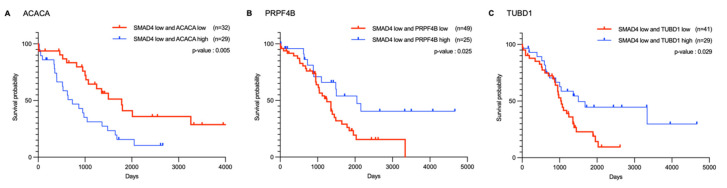
Kaplan–Meier survival analysis of three potential target genes in patients with low *SMAD4* expression. Survival curves comparing patients with ovarian cancer showing low *SMAD4* expression stratified by expression levels of (**A**) *ACACA*, (**B**) *PRPF4B*, and (**C**) *TUBD1*. For each gene, patients were categorized into high expression (blue lines) and low expression (red lines) groups. The x-axis represents the time in days, and the y-axis represents survival probability. All three genes showed significant differences in survival outcomes (*p* < 0.05) among patients with low *SMAD4* expression. The number of patients in each group is indicated in parentheses. Survival data for the remaining 17 potential target genes are presented in Figure S4.

Subsequently, Kaplan–Meier survival analysis and log-rank tests were performed to evaluate the association of these 20 genes with survival rates in patients with ovarian cancer (Figure S4). As the aim of this analysis was to identify genes that could overcome PARP inhibitor resistance in *SMAD4* mutant or low expression states, patients with ovarian cancer exhibiting low *SMAD4* expression were targeted. The analysis was conducted based on TCGA data by distinguishing patient groups with the highest and lowest 20% expression levels for each candidate gene. Consequently, three genes, *ACACA*, *PRPF4B*, and *TUBD1*, showed significant differences in survival rates (*p* ≤ 0.05) (Figure 9).

*PRPF4B* exhibited a distinct positive correlation with *SMAD4* with a Pearson correlation coefficient of 0.498. According to the survival analysis results, patient groups with low expressions of both *SMAD4* and *PRPF4B* showed significantly lower survival rates than those with high *PRPF4B* expression. Therefore, the co-expression of these two genes may influence patient prognosis. *TUBD1* expression showed a weak positive correlation with *SMAD4* expression (r = 0.374). Similar to *PRPF4B*, the survival analysis results indicated that patient groups with simultaneously low expression of *SMAD4* and *TUBD1* tended to have lower survival rates than those in the control group. Therefore, low *PRPF4B* and *TUBD1* expression in ovarian cancer may negatively affect the survival of patients with ovarian cancer having low *SMAD4* expression.

Conversely, although *ACACA* showed a distinct positive correlation with *SMAD4*, with a Pearson correlation coefficient of 0.470, survival analysis revealed contrasting findings. Within the low *SMAD4* expression group, patients with low *ACACA* expression showed higher survival rates than patients in the comparison group, suggesting that reduced *ACACA* expression could overcome the low survival rates caused by decreased *SMAD4* expression.

Based on a comprehensive evaluation using both Pearson correlation and survival analyses, 20 genes were found to have significant expression associations with *SMAD4* in ovarian cancer. Among these, *ACACA*, *PRPF4B*, and *TUBD1* were identified as potential target genes affecting survival rates, all showing significant positive correlations with *SMAD4*, indicating a tendency to increase or decrease in expression concordantly with *SMAD4* expression levels. Survival analysis revealed that, within the low *SMAD4* expression patient group, patients with low expression of both *PRPF4B* and *TUBD1* had significantly lower survival rates than patients with relatively high expression. These results suggest that *PRPF4B* and *TUBD1* may function as negative modifiers, which can decrease survival rates in low *SMAD4* expression states. *ACACA* also showed a positive correlation with *SMAD4* and shared expression patterns; however, in the low *SMAD4* expression patient group, low *ACACA* expression tended to significantly improve patient survival rates relative to those with high expression. This suggests that *ACACA* may aid in overcoming the decreased survival rate caused by low *SMAD4* expression.

Therefore, ACACA has potential as a therapeutic target for overcoming SMAD4-mediated resistance to PARP inhibitors. In particular, the prognostic value of *SMAD4* expression highlights the clinical relevance of this strategy, as tumors with low *SMAD4* expression may benefit from *ACACA* inhibition as a combinatorial approach. This interpretation is consistent with previous studies that have proposed therapeutic targets based on correlations between gene expression and patient survival, supporting the reliability of the present study interpretations [49,50].

## 3. Materials and Methods

### 3.1. Data Acquisition and Target Gene Selection

In this study, we utilized genomic drug sensitivity data to identify biomarkers for PARP inhibitors in ovarian cancer and additional candidate genes which could regulate these biomarkers. Using the ovarian cancer analysis of variance data obtained from the GDSC database (https://www.cancerrxgene.org/, accessed on 15 December 2023), we screened genomic features with *p*-value ≤ 0.05, to identify statistically significant candidate genes [51]. We confirmed that mutations in five candidate genes—*BRCA1*, *MLL2*, *NF1*, *SMAD4*, and *SMARCA4*— significantly altered the responses to five PARP inhibitor drugs (niraparib, olaparib, rucaparib, talazoparib, and veliparib). Results with *p* ≤ 0.05 were considered statistically significant (* *p* ≤ 0.05, ** *p* ≤ 0.01, *** *p* ≤ 0.001). Subsequently, we obtained gene mutation data for ovarian cancer cell lines from the DepMap portal (https://depmap.org/portal/, accessed on 15 December 2023); the detailed information is presented in Table 2 [30,31].

### 3.2. Drug Sensitivity Analysis

IC_50_ values were used to analyze the differences in sensitivity to PARP inhibitors based on the mutation status of each gene. IC_50_ is the half-maximal inhibitory concentration of a substance required to inhibit a biological process by 50% and is commonly used in method development for drug discovery [52]. For each of the five candidate genes, the mean and standard deviation of IC_50_ values for the PARP inhibitors (niraparib, olaparib, rucaparib, talazoparib, and veliparib) were calculated and compared between ovarian cancer cell lines with and without mutations.

### 3.3. Survival Analysis

Survival analysis was performed using patient survival data for ovarian cancer from TCGA database (https://www.cancer.gov/ccg/research/genome-sequencing/tcga, accessed on 15 December 2023) [53]. The survival data included information regarding the survival status of each patient and duration of survival. The gene expression levels of the patients were categorized for analysis based on the top and bottom 20% of patients with high and low expression, respectively. This top and bottom 20% cutoff was employed to maximize the contrast between groups and to exclude the intermediate range where the possibility of misclassification is high.

#### 3.3.1. Kaplan–Meier Estimator

Kaplan–Meier survival analysis is used for estimating the cumulative survival probability over time. The survival probability for each patient S(t) was cumulatively calculated based on the probability of death at a specific time t, and the Kaplan–Meier estimator was defined as follows:

(1) $$S\left(t\right)=\prod_{i:t_{i}\leq t}\left(1-\frac{d_{i}}{n_{i}}\right)\;,$$

where $t_{i}$ represents the time of death, $d_{i}$ is the number of patients who died at time point $t_{i}$, and $n_{i}$ is the number of patients alive at time point $t_{i}$. The survival probability at each time interval was calculated by multiplying the survival probability at the previous time point with the survival rate at the current time point.

#### 3.3.2. Log-Rank Test

The log-rank test is a survival analysis method testing the null hypothesis that there is no difference in the probability of event occurrence between two groups at all time points [54]. To verify whether the difference between two survival curves was statistically significant, we performed the log-rank test, where the test statistic Q was defined as

(2) $$Q=\sum_{i=1}^{k}\frac{{\left(O_{i}-E_{i}\right)}^{2}}{E_{i}},$$

where $O_{i}$ is the number of deaths observed in the i-th group and $E_{i}$ is the number of deaths expected in the i-th group based on the overall survival data. The test statistic Q follows a chi-square distribution with 1 degree of freedom ($X^{2}$); a *p*-value ≤ 0.05 indicated a significant difference.

### 3.4. Gene Dependency Score Analysis

The GDS, a quantitative value indicating how essential each gene is for the survival of cancer cells, was obtained from the DepMap portal, and the data were obtained using CRISPR-Cas9 technology [30,31]. GDS is expressed on a scale ranging from −1 to 1. Genes with a GDS close to −1 are considered highly essential for cell viability, while those with scores near 0 have minimal impact. Conversely, genes with a GDS close to 1 are typically associated with growth suppression or tumor suppressor functions.

An independent samples *t*-test was then conducted to compare GDS values for each gene according to *SMAD4* mutation status and expression levels. Consequently, genes showing significant differences were initially screened with *p*-value ≤ 0.05. In the second step, genes with mean GDS values < −0.5 in *SMAD4* mutant or low expression cell lines were further filtered as essential genes. This cutoff was applied as the criterion because genes within this range typically indicate essentiality for cell survival. Finally, genes whose mean GDS values in *SMAD4* mutant or low expression groups differed by at least 1.5-fold relative to their counterparts were selected through final screening, to identify potential targets influencing ovarian cancer cell viability.

### 3.5. Enrichment and Functional Analysis

#### 3.5.1. Gene Ontology Biological Process Analysis and Pathway Analysis

Enrichment analysis evaluates whether a set of genes of interest is significantly enriched for specific biological functions or pathways [32]. In this study, we performed GO biological process analysis based on the GO Biological Process 2025 gene set library and pathway analysis was performed using the Enrichr platform (https://maayanlab.cloud/Enrichr/, accessed on 15 December 2023) provided by the Ma’ayan Laboratory, based on the Kyoto Encyclopedia of Genes and Genomes (KEGG) 2021 pathway database [55,56]. Enrichr is a web-based tool that integrates various biological databases to analyze the enrichment of gene sets and provides prioritization through a combined score for each entry [57]. The formula for calculating the combined score is as follows:

(3) $$c=\log\left(p\right)\cdot z\;,$$

where c is the combined score, p is the *p*-value calculated using Fisher’s exact test, and z is the z-score calculated by evaluating the deviation from the expected ranking. Fisher’s exact test is a proportion test that assumes a binomial distribution and independence regarding the probability of any gene belonging to a particular set [57].

Researchers use various cutoffs for the combined score to evaluate the significance of gene set/functional enrichment findings in Enrichr. Generally, entries are identified by sorting the combined score in descending order after applying an adjusted *p*-value cutoff of 0.05–0.1 [58]. Similar criteria were used to sort the gene enrichment analysis results in this study.

#### 3.5.2. Hierarchical Clustering and Visualization

To visualize the functional similarities of the candidate genes, the results of GO biological process analysis are presented as bar graphs based on combined scores, and the results of pathway analysis are visualized as cluster maps combining heatmaps and dendrograms. To create cluster maps, we first constructed a binary matrix by extracting information regarding each biological pathway entry and the genes included in the respective pathways from the pathway analysis results. Subsequently, distances (similarities) between rows and columns were calculated based on the Euclidean distance, followed by hierarchical clustering using the average linkage method and visualization. This process was implemented in Python (version 3.10.0) using the SciPy (version 1.10.1) and Seaborn (version 0.12.2) libraries.

#### 3.5.3. Protein–Protein Interaction Network Analysis

To identify genes that could be functionally associated with *SMAD4*, a PPI network analysis was performed on selected candidate genes. The database and analytical tools provided by STRING (https://string-db.org/, accessed on 15 December 2023) were used to conduct an analysis based on the reported physical and functional interaction information in humans (*Homo sapiens*) [59]. STRING collects interaction information from various sources, including co-expression data, experimental results, curated databases such as KEGG or Reactome, and literature-based text mining, and provides a combined confidence score for each interaction. In this analysis, the significance of the interactions was determined based on the combined score, which assigned higher values when multiple types of evidence simultaneously supported the interaction [60].

### 3.6. Correlation Analysis

To evaluate the expression correlation between *SMAD4* and 51 potential candidate genes, Pearson correlation coefficient analysis was performed using gene expression data from the DepMap portal. Correlation between the expression values of each gene and *SMAD4* was calculated using Pearson’s correlation coefficient, defined as follows:

(4) $$r=\frac{\sum \left(X_{i}-\bar{X}\right)\left(Y_{i}-\bar{Y}\right)}{\sqrt{\sum {\left(X_{i}-\bar{X}\right)}^{2}}\cdot \sqrt{\sum {\left(Y_{i}-\bar{Y}\right)}^{2}}},$$

where $X_{i}$ and $Y_{i}$ represent the expression levels of *SMAD4* and the target gene, respectively, and $\bar{X}$ and $\bar{Y}$ indicate the mean expression levels of each gene. According to the absolute value criteria for the Pearson correlation coefficient, while interpretations may vary, generally 0 <|r|≤ 0.2 indicates a very weak correlation, 0.2 <|r|≤ 0.4 indicates a weak correlation, 0.4 <|r|≤ 0.65 indicates a moderate correlation, 0.65 <|r|≤ 0.85 indicates a strong correlation, and 0.85 <|r|≤ 1 indicates a very strong correlation. Based on these criteria, genes with |r|≥ 0.35 were considered to have a significant correlation, and this threshold was used for initial screening to select candidate genes for survival analysis.

## 4. Conclusions

In the present study, we identified biomarkers for PARP inhibitor sensitivity and resistance in ovarian cancer through genomic drug sensitivity data and biological big data analysis (Figure 1). Drug sensitivity analysis using GDSC data revealed that *BRCA1, MLL2, NF1,* and *SMARCA4* mutations enhance sensitivity to PARP inhibitors, suggesting synthetic lethal relationships with *PARP1* and their potential as predictive biomarkers. Conversely, *SMAD4* mutations increased resistance to PARP inhibitors in ovarian cancer cell lines, and survival analysis using TCGA data confirmed that patients with low *SMAD4* expression exhibit significantly poor survival rates. These findings highlight *SMAD4* as a major biomarker, potentially involving a DD-type synthetic rescue relationship with *PARP1*. However, the approach adopted in this study has not been experimentally validated and will require targeted in vitro/in vivo functional studies.

Through GDS analysis of DepMap data, we identified 51 potential target genes related to SMAD4-mediated resistance. Enrichment analysis revealed their involvement in cellular stress responses, lipid biosynthesis, PI3K–Akt and MAPK signaling, transcriptional regulation, and energy metabolism. PPI network analysis confirmed strong functional associations between *SMAD4* and *USP9X, YAP1,* and *MAPK1*. Gene expression correlation and survival analysis identified three genes—*ACACA*, *PRPF4B*, and *TUBD1*—as significantly influencing survival rates. Notably, decreased *ACACA* expression in patients with low *SMAD4* expression was associated with improved survival, suggesting its potential for overcoming *SMAD4*-mediated PARP inhibitor resistance. In this study, the clinical application of *SMAD4* as a biomarker of resistance to PARP inhibitors is possibly specific to ovarian cancer.

This study also has certain limitations, as it relies on computational predictions derived from multiple datasets and does not incorporate direct functional validation. Moreover, the cell line and TCGA data analyzed here may not fully capture the complexity of individual patient responses to treatment.

Taken together, these findings contribute to personalized treatment strategies by providing biomarkers for predicting PARP inhibitor efficacy and identifying potential targets for overcoming resistance in patients with ovarian cancer, while underscoring the need for further experimental and clinical validation.

## Figures and Tables

**Table 1:** IC_50_ values of five PARP inhibitors in mutant and wild-type ovarian cancer cell lines for *BRCA1*, *MLL2*, *NF1*, *SMAD4*, and *SMARCA4*. IC_50_ values are presented as mean ± standard deviation (Mean ± SD) and expressed in μM (micromolar).

Gene	Drug	Wild-Type (n)	IC_50_ (WT*) ± SD	Mutant (n)	IC_50_ (MT^†^) ± SD
* BRCA1 *	Niraparib	24	149.2 ± 125.8	4	36.8 ± 14.1
	Olaparib	29	297.3 ± 495.8	4	77.9 ± 84.7
	Rucaparib	27	163.3 ± 344.9	4	28.1 ± 10.2
	Talazoparib	29	247.2 ± 688.1	4	12.2 ± 15.7
	Veliparib	28	505.3 ± 677.0	4	280.7 ± 240.6
* MLL2 *	Niraparib	26	141.4 ± 123.8	2	25.5 ± 3.5
	Olaparib	30	291.0 ± 473.7	3	67.8 ± 43.4
	Rucaparib	26	165.4 ± 351.6	3	30.7 ± 15.3
	Talazoparib	30	239.3 ± 677.5	3	12.5 ± 16.7
	Veliparib	29	516.5 ± 661.2	3	97.3 ± 49.3
* NF1 *	Niraparib	24	149.4 ± 125.4	4	35.5 ± 26.7
	Olaparib	29	288.9 ± 481.3	4	138.9 ± 163.2
	Rucaparib	27	162.1 ± 345.3	4	35.8 ± 2.1
	Talazoparib	28	233.0 ± 700.9	4	13.4 ± 12.3
	Veliparib	28	517.2 ± 676.5	4	197.5 ± 65.7
* SMAD4 *	Niraparib	25	124.0 ± 117.8	3	209.3 ± 166.9
	Olaparib	29	171.9 ± 148.0	4	987.1 ± 1110.1
	Rucaparib	27	81.3 ± 58.2	4	581.5 ± 855.7
	Talazoparib	29	123.1 ± 337.1	4	911.5 ± 1641.8
	Veliparib	28	331.5 ± 211.7	4	1497.0 ± 1501.3
* SMARCA4 *	Niraparib	25	144.2 ± 125.5	3	40.6 ± 24.9
	Olaparib	30	291.7 ± 473.5	3	61.6 ± 10.3
	Rucaparib	28	154.2 ± 340.7	3	67.8 ± 28.6
	Talazoparib	30	240.3 ± 677.2	3	2.8 ± 1.3
	Veliparib	29	503.4 ± 667.3	3	223.8 ± 148.1

WT*; Wild-type, MT^†^; Mutant.

**Table 2:** Classification of ovarian cancer cell lines based on *SMAD4* status. Ovarian cancer cell lines were classified according to both *SMAD4* mutation status based on genomic analysis from the Genomics of Drug Sensitivity in Cancer (GDSC) databases and *SMAD4* expression levels based on the genomic analysis from DepMap databases. For expression-based classification, cell lines were categorized using the highest and lowest 30% of *SMAD4* expression levels as the criteria.

Group	Cell Lines
Wild-type	PEO1, FU-OV-1, DOV13, OAW42, OVK18, UWB1.289, Caov-3, OVTOKO, OV-17R, OVCA433, OVCAR-4, JHOS-3, Caov-4, TOV-21G, OAW28, JHOS-4, SK-OV-3, OVCAR-8, OV-56, OVCAR-3, OVMIU, OVCA420, TOV-112D, OC-314, OVCAR-5, JHOS-2, OVISE, Hey, OV-7
*SMAD4* Mutant	IGROV-1, OVKATE, SW626, OV-90
High *SMAD4* Expression	OAW28, SK-OV-3, JHOS-4, JHOM-1, JHOC-5, NZOV9, PEA1, A2780, BIN-67, COV504, TOV-112D, OC-316, OVCAR-8, FU-OV-1, COV434, OVK18, OV-7, OVSAHO
Low *SMAD4* Expression	JHOM-2B, OVTOKO, MCAS, OCI-P5x, 59M, OAW42, UWB1.289, OCI-C4P, OCI-C5x, OVKATE, COV413A, OVCAR-5, OVCAR-4, PEO1, OV-90, PEO4, OVMANA, RMUG-S

**Table 3:** Gene dependency scores (GDS) of potential target genes in ovarian cancer cell lines based on *SMAD4* mutation status. List of 33 genes showing significantly different GDS values between *SMAD4* mutant and wild-type cell lines. The numerical values in the table represent the average GDS values for each gene in the respective cell line groups, with *p*-values indicating the statistical significance of differences between groups. Negative GDS values indicate greater dependency, suggesting that these genes are essential for cell survival in the respective *SMAD4* context.

Gene	*p*-Value	GDS in Mutant Cell Lines	GDS in Wild-Type Cell Lines
* MAPK1 *	0.0004	−0.9643	−0.1284
* INTS6 *	0.0007	−1.7723	−0.6206
* TTC7A *	0.0008	−0.5756	−0.1820
* MTA2 *	0.0018	−0.8225	−0.2730
* SOX2 *	0.0034	−0.5008	−0.1705
* MAP2K1 *	0.0053	−0.5004	−0.1118
* PPP2R2A *	0.0060	−0.5252	−0.1287
* RPL28 *	0.0083	−1.3475	−0.8033
* TYRO3 *	0.0093	−0.6591	−0.3882
* KAT2A *	0.0093	−0.5809	−0.2899
* ARHGEF5 *	0.0095	−0.6281	−0.3619
* DOCK5 *	0.0098	−0.5625	−0.1139
* HSPA8 *	0.0109	−1.4931	−0.6527
* HIC2 *	0.0121	−0.5542	−0.0774
* PKN2 *	0.0124	−0.8481	−0.4446
* SCARF2 *	0.0145	−0.5807	−0.1586
* MED15 *	0.0159	−0.6711	−0.1594
* ARF6 *	0.0171	−0.6359	−0.2516
* CDK4 *	0.0186	−1.4800	−0.6004
* TIMM29 *	0.0197	−0.8811	−0.5807
* HEXIM1 *	0.0218	−0.6848	−0.3736
* YAP1 *	0.0261	−1.2623	−0.5317
* ASH2L *	0.0264	−0.6810	−0.3158
* TUBD1 *	0.0283	−0.7341	−0.4012
* H2AW *	0.0299	−0.5787	−0.3424
* EIF1 *	0.0357	−1.6192	−1.0654
* PPP2CA *	0.0452	−1.5073	−0.7478
* PRPF4B *	0.0455	−0.9152	−0.6036
* ACACA *	0.0462	−0.6231	−0.3011
* SAP130 *	0.0465	−0.7302	−0.4637
* KRTAP9-6 *	0.0468	−0.6083	−0.3868
* ILK *	0.0478	−0.8788	−0.4457
* FASN *	0.0497	−0.5177	−0.2311

**Table 4:** GDS of potential target genes in ovarian cancer cell lines based on *SMAD4* expression levels. List of 18 genes displaying significantly different GDS values between cell lines with low and high *SMAD4* expression levels. The numerical values in the table represent the average GDS values for each gene in the respective cell line groups, with *p*-values indicating the statistical significance of differences between groups. Negative GDS values indicate greater dependency, suggesting these genes are essential for cell survival in the respective *SMAD4* context.

Gene	*p*-Value	GDS in Low Expression Cell Lines	GDS in High Expression Cell Lines
* SMARCA4 *	0.0019	−0.6337	−0.2506
* WNK1 *	0.0025	−0.7793	−0.4229
* RAB6A *	0.0044	−0.5010	−0.1044
* VPS4A *	0.0047	−0.7433	−0.3425
* GFPT1 *	0.0124	−0.7171	−0.4088
* ARHGAP29 *	0.0129	−0.5395	−0.1669
* RIC1 *	0.0135	−0.7341	−0.3076
* TUBB4B *	0.0147	−0.5456	−0.3078
* WWTR1 *	0.0152	−0.5619	−0.3043
* KLF5 *	0.0154	−0.6163	−0.2499
* CAND1 *	0.0157	−0.5319	−0.2209
* TRPM7 *	0.0199	−0.7870	−0.4315
* SMARCB1 *	0.0240	−0.8087	−0.4964
* YRDC *	0.0340	−2.2461	−1.4869
* TFRC *	0.0342	−0.9311	−0.5985
* EFR3A *	0.0422	−0.6048	−0.3122
* SLC2A1 *	0.0458	−0.5958	−0.3070
* USP9X *	0.0491	−0.6929	−0.3983

## Data Availability

The datasets used and/or analyzed during the current study are available from the corresponding author on reasonable request.

## Supplementary Materials

The supporting information can be downloaded at https://www.mdpi.com/article/10.3390/ijms26199530/s1.

## Abbreviations

The following abbreviations are used in this manuscript:

CI	Confidence Interval
DepMap	Cancer Dependency Map
FDA	Food and Drug Administration
GDSC	The Genomics of Drug Sensitivity in Cancer
GO	Gene Ontology
HR	Hazard Ratio
HRD	Homologous Recombination Deficiency
PARP	Poly (ADP-ribose) polymerase
KEGG	Kyoto Encyclopedia of Genes and Genomes
MST	Median Survival Time
PPI	Protein–Protein Interaction
TCGA	The Cancer Genome Atlas
