# Supplementary material for: Identification of Anticancer Targets in Ovarian Cancer Using Genomic Drug Sensitivity Data
Source: Int J Mol Sci. 2025 Sep 29;26(19):9530. doi: 10.3390/ijms26199530 (PMC12524686; doi:10.3390/ijms26199530)
Supplement: Supplementary file 1 [file ijms-26-09530-s001.zip › ijms-3852319-supplementary.pdf]

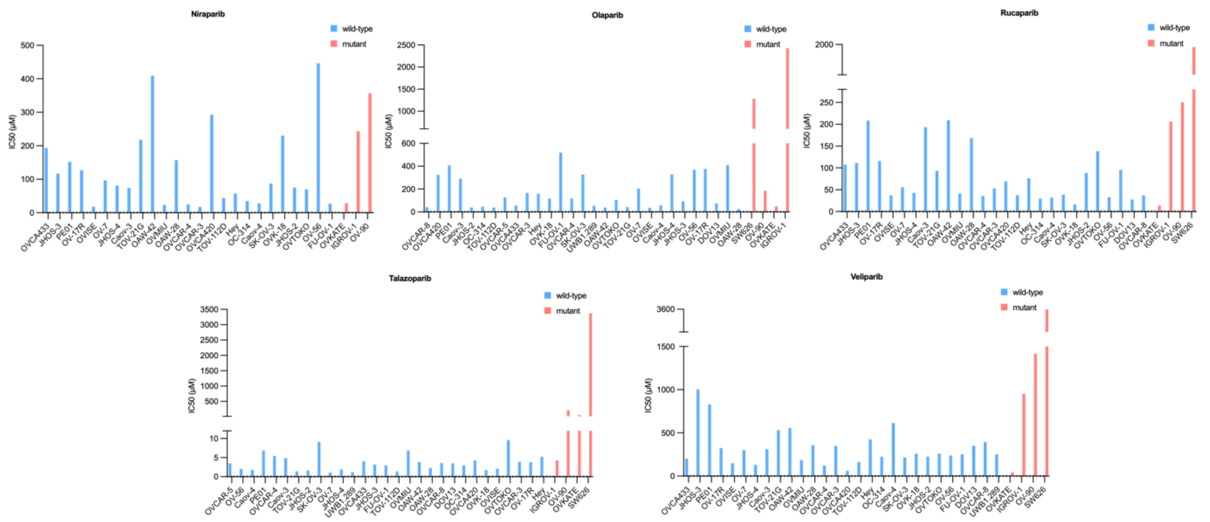

Supplementary Figure S1 Individual IC_{50} values of PARP inhibitors across ovarian cancer cell lines. Bar graphs showing the IC_{50} values (μM) of five PARP inhibitors (Niraparib, Olaparib, Rucaparib, Talazoparib, and Veliparib) for each ovarian cancer cell line. Blue bars represent wild-type cell lines and red bars represent *SMAD4* mutant cell lines. The y-axis indicates IC_{50} values, and the x-axis presents individual ovarian cancer cell lines.

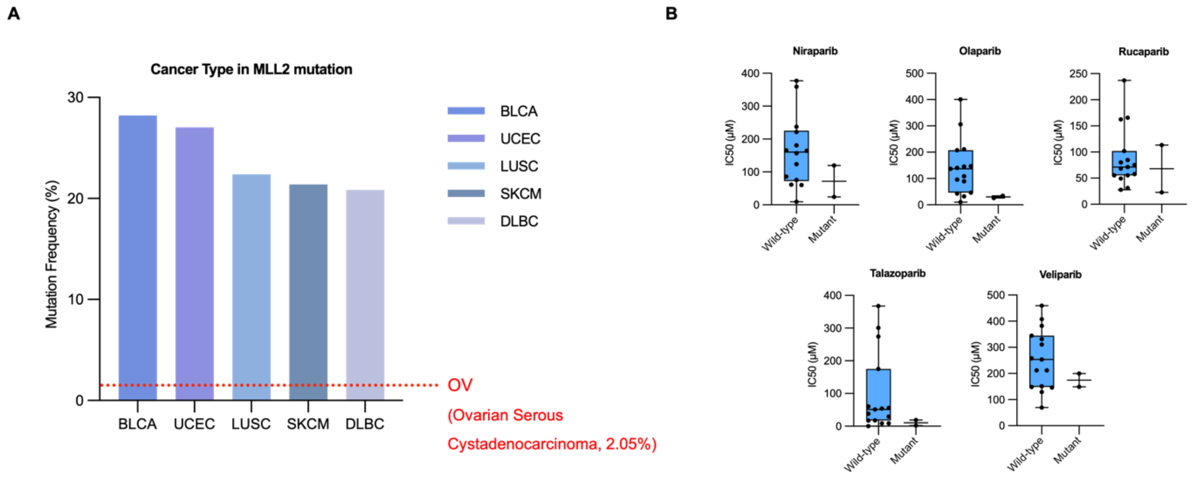

Supplementary Figure S2 *MLL2* mutation frequency across cancer types and PARP inhibitor sensitivity analysis in bladder cancer. (**A**) Bar graph showing the following top five cancer types with the highest *MLL2* mutation frequencies: Bladder Urothelial Carcinoma (BLCA, 28.22%), Uterine Corpus Endometrial Carcinoma (UCEC, 27.03%), Lung Squamous Cell Carcinoma (LUSC, 22.38%), Skin Cutaneous Melanoma (SKCM, 21.4%), and Diffuse Large B-Cell Lymphoma (DLBC, 20.83%). The red dotted line indicates the mutation frequency in Ovarian Serous Cystadenocarcinoma (OV, 2.05%). Data were obtained from cBioPortal for Cancer Genomics. (**B**) Drug Sensitivity analysis comparing the IC_{50} values of five PARP inhibitors (Niraparib, Olaparib, Rucaparib, Talazoparib, and Veliparib) between *MLL2* wild-type and mutant bladder cancer (BLCA) cell lines. Box plots show the distribution of IC_{50} values (μM) for each inhibitor.

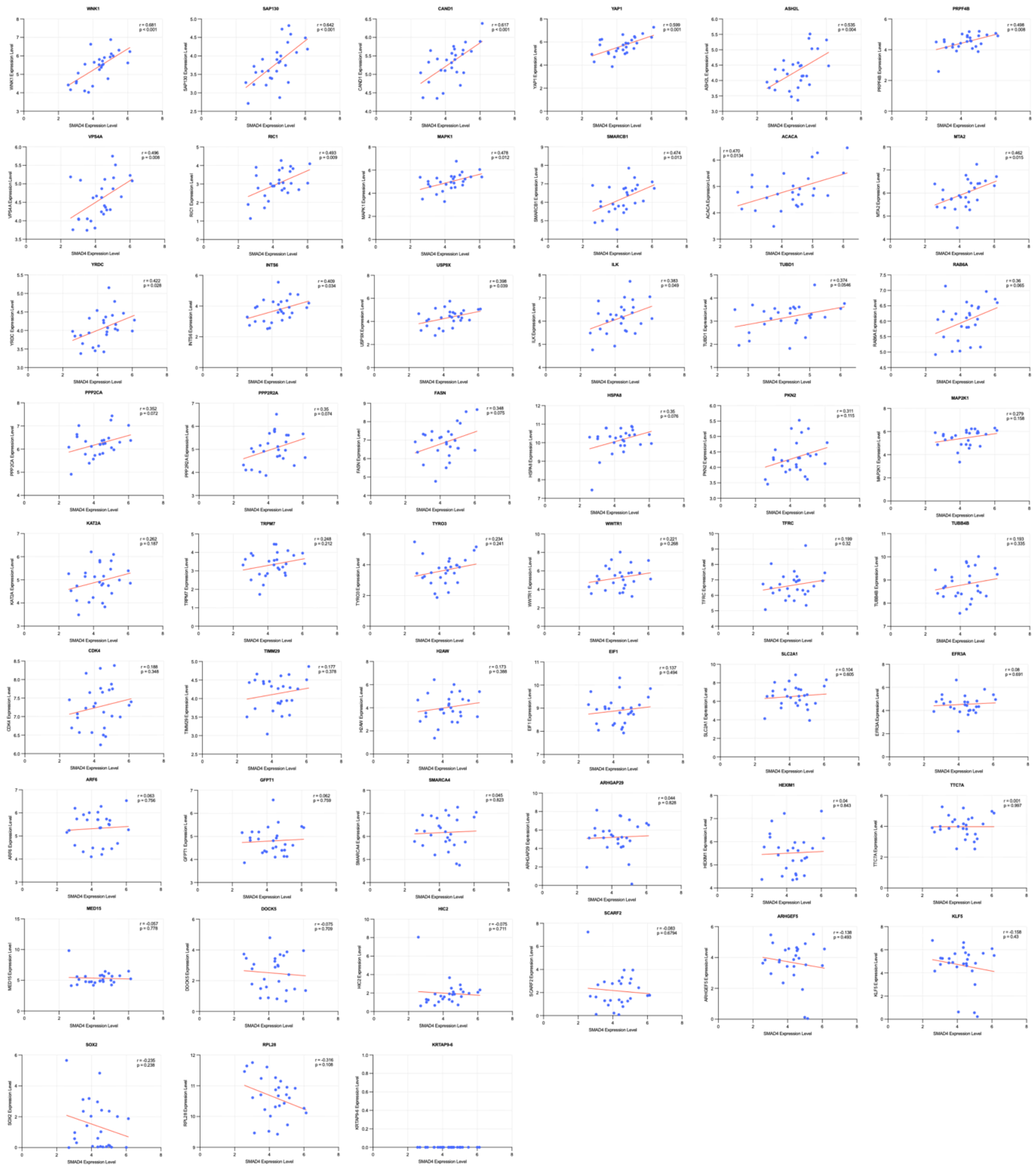

Supplementary Figure S3 Gene expression correlation analysis between *SMAD4* and 51 screened candidate genes in ovarian cancer cell lines. Scatter plots displaying the correlation of gene expression levels between *SMAD4* (x-axis) and each of the 51 candidate genes (y-axis) identified from previous screening. Each dot represents an individual ovarian cancer cell line. Blue dots indicate the expression data points from each cell line.

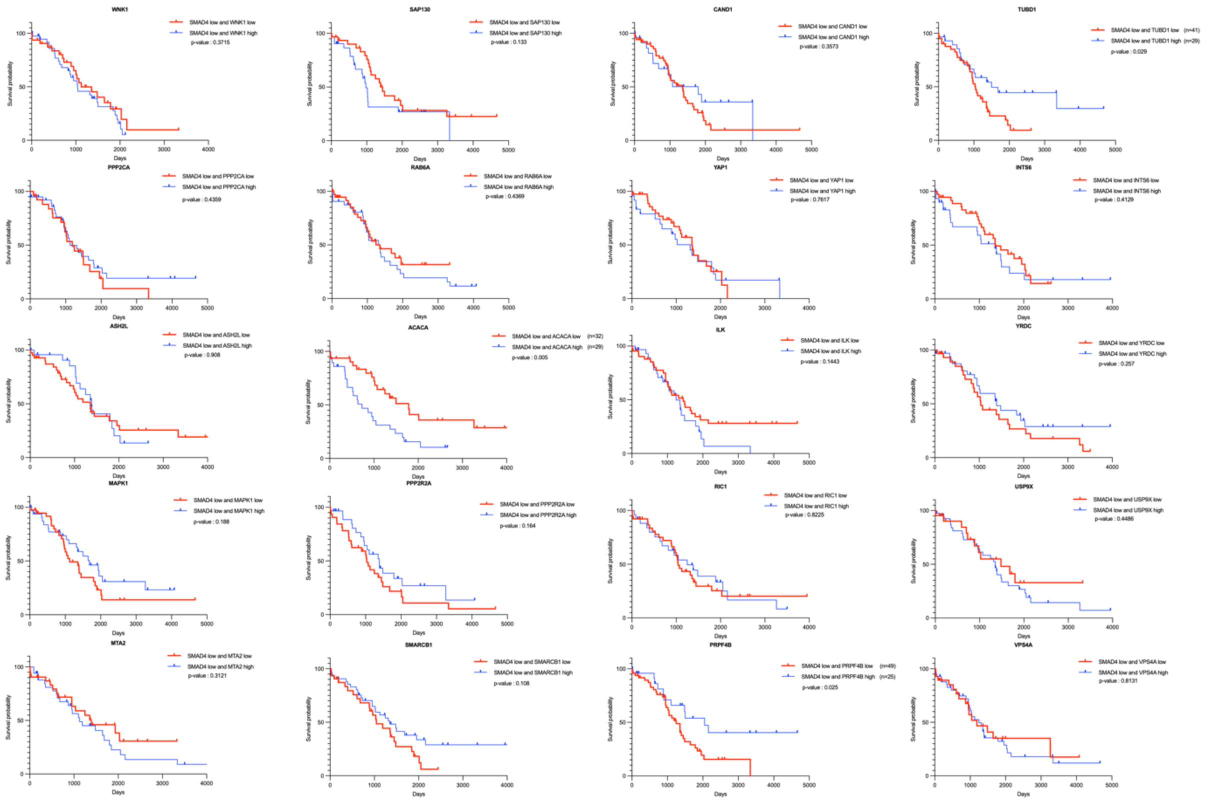

Supplementary Figure S4 Kaplan–Meier survival analysis of 20 potential target genes in patients with low *SMAD4* expression. Survival curves comparing patients with ovarian cancer showing low *SMAD4* expression stratified by expression levels (high and low) of 20 potential target genes screened through correlation analysis. For each gene, patients with low *SMAD4* expression were categorized into high target gene expression (blue lines) and low target gene expression (red lines) groups. The x-axis represents the time in days, and the y-axis represents survival probability. P-values for each analysis are indicated in the respective plots.

A

Cancer type list
Acute lymphoblastic leukemia (ALL)
Acute myeloid leukemia (LAML)
Adrenocortical carcinoma (ACC)
Bladder urothelial carcinoma (BLCA)
Brain lower grade glioma (LGG)
Breast invasive carcinoma (BRCA)
Cervical squamous cell carcinoma and endocervical adenocarcinoma (CESC)
Chronic lymphocytic leukemia (CLL)
Chronic myelogenous leukemia (LCML)
Colon and rectum adenocarcinoma (COAD/READ) (COREAD)
Esophageal carcinoma (ESCA)
Glioblastoma multiforme (GBM)
Head and neck squamous cell carcinoma (HNSC)
Kidney renal clear cell carcinoma (KIRC)
Liver hepatocellular carcinoma (LIHC)
Lung adenocarcinoma (LUAD)
Lung squamous cell carcinoma (LUSC)
Lymphoid neoplasm diffuse large B-cell lymphoma (DLBC)
Medulloblastoma (MB)
Mesothelioma (MESO)
Multiple myeloma (MM)
Neuroblastoma (NB)
Ovarian serous cystadenocarcinoma (OV)
Pancreatic adenocarcinoma (PAAD)
Prostate adenocarcinoma (PRAD)
Skin cutaneous melanoma (SKCM)
Small cell lung cancer (SCLC)
Stomach adenocarcinoma (STAD)
Thyroid carcinoma (THCA)
Uterine corpus endometrial carcinoma (UCEC)

B

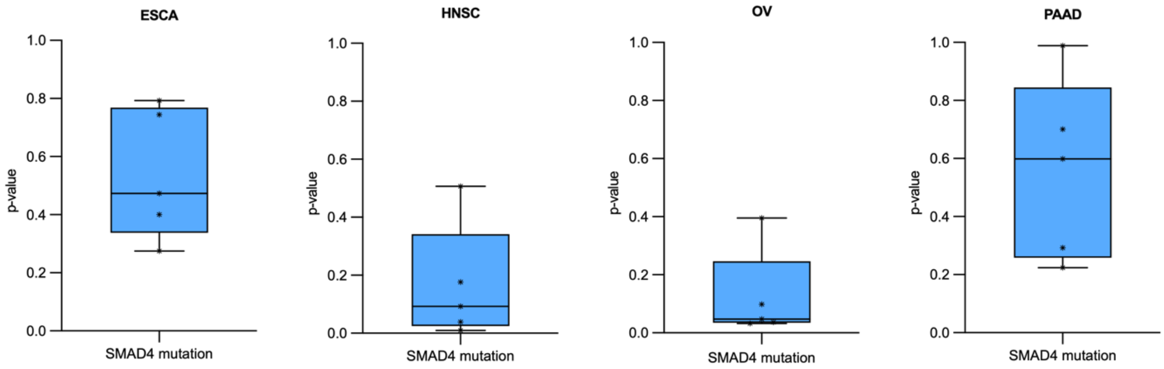

Supplementary Figure S5 Analysis of *SMAD4* mutations across different cancer types. (**A**) List of 30 cancer types investigated for *SMAD4* mutation status using the GDSC database. (**B**) Box plots showing p-values for *SMAD4* mutation significance in the following four cancer types exhibiting *SMAD4* mutations: Esophageal Carcinoma (ESCA, p=0.474), Head and Neck Squamous Cell Carcinoma (HNSC, p=0.092), Ovarian Serous Cystadenocarcinoma (OV, p=0.047), and Pancreatic Adenocarcinoma (PAAD, p=0.599). Only OV showed statistically significant results (p<0.05), suggesting that the findings regarding *SMAD4* mutation as a resistance biomarker are potentially specific to ovarian cancer.

Supplementary Table S1 Survival analysis results of patients with ovarian cancer stratified according to gene expression levels for *BRCA1*, *MLL2*, *NF1*, *SMAD4*, and *SMARCA4*. The high and low expression groups represent patients in the top and bottom 20% of gene expression, respectively. X^2 (df=1) denotes the log-rank test statistic with one degree of freedom, HR represents hazard ratio, and CI indicates confidence interval.

Gene	Group	n	Median Survival (day)	$\boldsymbol{X^2}$ (df=1)	p-value	HR (95% CI)
BRCA1	High Expression	75	1189	0.132	0.7168	1.079 (0.7153–1.627)
	Low Expression	74	1348			0.9270 (0.6146–1.398)
MLL2	High Expression	74	1446	0.002	0.9672	0.9915 (0.6574–1.495)
	Low Expression	75	1359			1.009 (0.6687–1.521)
NF1	High Expression	75	1314	2.173	0.1404	1.352 (0.9035–2.022)
	Low Expression	75	1516			0.7398 (0.4945–1.107)
SMAD4	High Expression	75	1720	5.578	0.0182	0.6087 (0.4020–0.9218)
	Low Expression	75	1123			1.643 (1.085–2.488)
SMARCA4	High Expression	75	1187	0.020	0.8882	1.031 (0.6731–1.578)
	Low Expression	74	1366			0.9702 (0.6336–1.486)

Supplementary Table S2 Raw gene expression data used in the correlation analysis presented in Figure 8. Data were collected from ovarian cancer cell lines that contained complete expression data for both *SMAD4* and target genes. Target genes are arranged in descending order of Pearson correlation coefficients as presented in Figure 8.

Target gene	Cell line	*SMAD4* expression	Target gene expression
WNK1	JHOS-2	4.520422	5.491532
WNK1	CAOV-3	4.654779	5.906891
WNK1	OVISE	3.862947	6.629211
WNK1	SW626	4.350497	5.522935
WNK1	OVCAR-4	3.028569	4.529821
WNK1	IGROV-1	4.36807	5.256256
WNK1	OVCAR-5	3.037382	4.615887
WNK1	OV-17R	3.97728	4.356144
WNK1	OVTOKO	3.739848	4.022368
WNK1	TOV-112D	5.159064	6.002928
WNK1	OVK18	4.987321	5.938286
WNK1	SK-OV-3	6.0054	5.61912
WNK1	UWB1.289	3.514753	4.101818
WNK1	OVKATE	3.140779	5.06695
WNK1	OVCAR-8	5.051807	6.112075
WNK1	TOV-21G	4.543496	5.722193
WNK1	OV-56	4.361768	5.80684
WNK1	OAW28	6.127221	6.232661
WNK1	PEO1	2.70044	4.164304
WNK1	OAW42	3.529821	5.545968
WNK1	OV-90	2.568032	4.42425
WNK1	FU-OV-1	5.018812	6.879706
WNK1	OV-7	4.860963	4.766065
WNK1	JHOS-4	5.495056	6.312338
WNK1	OVCAR-3	4.474436	5.377124
WNK1	OC-314	4.066089	5.653633
WNK1	CAOV-4	4.58376	5.542877
SAP130	JHOS-2	4.520422	2.871844
SAP130	CAOV-3	4.654779	4.726286
SAP130	OVISE	3.862947	3.594549
SAP130	SW626	4.350497	3.586164
SAP130	OVCAR-4	3.028569	3.226509
SAP130	IGROV-1	4.36807	4.097611
SAP130	OVCAR-5	3.037382	3.716991
SAP130	OV-17R	3.97728	3.404631
SAP130	OVTOKO	3.739848	3.209453
SAP130	TOV-112D	5.159064	4.589164
SAP130	OVK18	4.987321	4.36807

SAP130	SK-OV-3	6.0054	4.488644
SAP130	UWB1.289	3.514753	3.582556
SAP130	OVKATE	3.140779	3.553361
SAP130	OVCAR-8	5.051807	4.82222
SAP130	TOV-21G	4.543496	3.852998
SAP130	OV-56	4.361768	3.900142
SAP130	OAW28	6.127221	4.185074
SAP130	PEO1	2.70044	2.713696
SAP130	OAW42	3.529821	3.908813
SAP130	OV-90	2.568032	3.277985
SAP130	FU-OV-1	5.018812	3.28244
SAP130	OV-7	4.860963	3.745237
SAP130	JHOS-4	5.495056	4.094236
SAP130	OVCAR-3	4.474436	3.82069
SAP130	OC-314	4.066089	3.389567
SAP130	CAOV-4	4.58376	3.999098
CAND1	JHOS-2	4.520422	5.686781
CAND1	CAOV-3	4.654779	5.060912
CAND1	OVISE	3.862947	4.484783
CAND1	SW626	4.350497	5.314697
CAND1	OVCAR-4	3.028569	5.330917
CAND1	IGROV-1	4.36807	5.569552
CAND1	OVCAR-5	3.037382	4.788164
CAND1	OV-17R	3.97728	5.100137
CAND1	OVTOKO	3.739848	5.111866
CAND1	TOV-112D	5.159064	5.874059
CAND1	OVK18	4.987321	5.549669
CAND1	SK-OV-3	6.0054	5.891176
CAND1	UWB1.289	3.514753	4.348374
CAND1	OVKATE	3.140779	5.643856
CAND1	OVCAR-8	5.051807	5.045705
CAND1	TOV-21G	4.543496	5.400879
CAND1	OV-56	4.361768	5.178715
CAND1	OAW28	6.127221	6.380591
CAND1	PEO1	2.70044	4.367371
CAND1	OAW42	3.529821	5.417515
CAND1	OV-90	2.568032	5.042207
CAND1	FU-OV-1	5.018812	5.657926
CAND1	OV-7	4.860963	4.702658
CAND1	JHOS-4	5.495056	5.62293
CAND1	OVCAR-3	4.474436	5.759955
CAND1	OC-314	4.066089	5.390943
CAND1	CAOV-4	4.58376	5.517591
YAP1	JHOS-2	4.520422	5.918625

YAP1	CAOV-3	4.654779	5.883865
YAP1	OVISE	3.862947	4.70044
YAP1	SW626	4.350497	6.652773
YAP1	OVCAR-4	3.028569	5.759955
YAP1	IGROV-1	4.36807	5.749802
YAP1	OVCAR-5	3.037382	6.197512
YAP1	OV-17R	3.97728	5.231509
YAP1	OVTOKO	3.739848	3.870858
YAP1	TOV-112D	5.159064	5.908333
YAP1	OVK18	4.987321	5.444932
YAP1	SK-OV-3	6.0054	6.411087
YAP1	UWB1.289	3.514753	5.102238
YAP1	OVKATE	3.140779	6.239551
YAP1	OVCAR-8	5.051807	6.403609
YAP1	TOV-21G	4.543496	5.565293
YAP1	OV-56	4.361768	5.324451
YAP1	OAW28	6.127221	7.283181
YAP1	PEO1	2.70044	4.283922
YAP1	OAW42	3.529821	4.995937
YAP1	OV-90	2.568032	4.938756
YAP1	FU-OV-1	5.018812	6.478325
YAP1	OV-7	4.860963	6.172728
YAP1	JHOS-4	5.495056	6.737957
YAP1	OVCAR-3	4.474436	4.981853
YAP1	OC-314	4.066089	5.448571
YAP1	CAOV-4	4.58376	5.082362
ASH2L	JHOS-2	4.520422	4.003602
ASH2L	CAOV-3	4.654779	4.15056
ASH2L	OVISE	3.862947	3.650765
ASH2L	SW626	4.350497	3.361768
ASH2L	OVCAR-4	3.028569	3.690417
ASH2L	IGROV-1	4.36807	4.576522
ASH2L	OVCAR-5	3.037382	4.361066
ASH2L	OV-17R	3.97728	3.476382
ASH2L	OVTOKO	3.739848	4.213347
ASH2L	TOV-112D	5.159064	5.034304
ASH2L	OVK18	4.987321	5.37121
ASH2L	SK-OV-3	6.0054	5.319401
ASH2L	UWB1.289	3.514753	4.341274
ASH2L	OVKATE	3.140779	3.973611
ASH2L	OVCAR-8	5.051807	5.513175
ASH2L	TOV-21G	4.543496	4.492494
ASH2L	OV-56	4.361768	3.769772
ASH2L	OAW28	6.127221	4.475085

ASH2L	PEO1	2.70044	3.764474
ASH2L	OAW42	3.529821	4.651913
ASH2L	OV-90	2.568032	3.948601
ASH2L	FU-OV-1	5.018812	3.866908
ASH2L	OV-7	4.860963	4.146492
ASH2L	JHOS-4	5.495056	5.036064
ASH2L	OVCAR-3	4.474436	4.472488
ASH2L	OC-314	4.066089	4.521051
ASH2L	CAOV-4	4.58376	4.144862
PRPF4B	TOV-21G	4.543496	4.593354
PRPF4B	OVTOKO	3.739848	3.91934
PRPF4B	OVK18	4.987321	3.877744
PRPF4B	OV-56	4.361768	4.835419
PRPF4B	OVKATE	3.140779	4.141596
PRPF4B	CAOV-3	4.654779	4.084915
PRPF4B	OVCAR-8	5.051807	5.199672
PRPF4B	OAW28	6.127221	4.89724
PRPF4B	SW626	4.350497	4.535431
PRPF4B	OV-7	4.860963	4.211012
PRPF4B	SK-OV-3	6.0054	5.06996
PRPF4B	IGROV-1	4.36807	4.927896
PRPF4B	TOV-112D	5.159064	4.386811
PRPF4B	OVCAR-3	4.474436	5.178715
PRPF4B	OV-90	2.568032	4.502712
PRPF4B	OVCAR-4	3.028569	4.654779
PRPF4B	OAW42	3.529821	4.357552
PRPF4B	OVCAR-5	3.037382	4.519793
PRPF4B	OVISE	3.862947	4.219556
PRPF4B	OV-17R	3.97728	4.420887
PRPF4B	UWB1.289	3.514753	4.160275
PRPF4B	OC-314	4.066089	5.024586
PRPF4B	JHOS-2	4.520422	4.77663
PRPF4B	JHOS-4	5.495056	4.965323
PRPF4B	CAOV-4	4.58376	4.563768
PRPF4B	FU-OV-1	5.018812	4.90641
PRPF4B	PEO1	2.70044	2.589763
VPS4A	JHOS-2	4.520422	4.408712
VPS4A	CAOV-3	4.654779	4.292045
VPS4A	OVISE	3.862947	4.621173
VPS4A	SW626	4.350497	4.242603
VPS4A	OVCAR-4	3.028569	4.050502
VPS4A	IGROV-1	4.36807	4.869378
VPS4A	OVCAR-5	3.037382	4.033863
VPS4A	OV-17R	3.97728	3.802193

VPS4A	OVTOKO	3.739848	3.991862
VPS4A	TOV-112D	5.159064	5.514438
VPS4A	OVK18	4.987321	5.750607
VPS4A	SK-OV-3	6.0054	5.227279
VPS4A	UWB1.289	3.514753	3.743084
VPS4A	OVKATE	3.140779	5.099295
VPS4A	OVCAR-8	5.051807	5.163901
VPS4A	TOV-21G	4.543496	5.122673
VPS4A	OV-56	4.361768	4.630522
VPS4A	OAW28	6.127221	5.080231
VPS4A	PEO1	2.70044	3.75809
VPS4A	OAW42	3.529821	4.05398
VPS4A	OV-90	2.568032	5.187847
VPS4A	FU-OV-1	5.018812	4.86839
VPS4A	OV-7	4.860963	4.301588
VPS4A	JHOS-4	5.495056	4.651913
VPS4A	OVCAR-3	4.474436	4.179511
VPS4A	OC-314	4.066089	4.670727
VPS4A	CAOV-4	4.58376	4.365273
RIC1	JHOS-2	4.520422	2.664483
RIC1	CAOV-3	4.654779	2.761285
RIC1	OVISE	3.862947	3.01614
RIC1	SW626	4.350497	3.039138
RIC1	OVCAR-4	3.028569	3.902074
RIC1	IGROV-1	4.36807	3.751678
RIC1	OVCAR-5	3.037382	3.454176
RIC1	OV-17R	3.97728	2.887525
RIC1	OVTOKO	3.739848	2.06695
RIC1	TOV-112D	5.159064	3.83289
RIC1	OVK18	4.987321	3.676944
RIC1	SK-OV-3	6.0054	3.047887
RIC1	UWB1.289	3.514753	1.709291
RIC1	OVKATE	3.140779	2.78031
RIC1	OVCAR-8	5.051807	3.037382
RIC1	TOV-21G	4.543496	3.869871
RIC1	OV-56	4.361768	3.221877
RIC1	OAW28	6.127221	4.0917
RIC1	PEO1	2.70044	1.137504
RIC1	OAW42	3.529821	2.370164
RIC1	OV-90	2.568032	1.895303
RIC1	FU-OV-1	5.018812	3.957915
RIC1	OV-7	4.860963	2.526069
RIC1	JHOS-4	5.495056	2.693766
RIC1	OVCAR-3	4.474436	4.266787

RIC1	OC-314	4.066089	2.893362
RIC1	CAOV-4	4.58376	3.068671
MAPK1	TOV-21G	4.543496	5.429951
MAPK1	OVTOKO	3.739848	4.971314
MAPK1	OVK18	4.987321	5.833902
MAPK1	OV-56	4.361768	4.465322
MAPK1	OVKATE	3.140779	4.582556
MAPK1	CAOV-3	4.654779	6.769904
MAPK1	OVCAR-8	5.051807	5.01614
MAPK1	OAW28	6.127221	5.408712
MAPK1	SW626	4.350497	4.959306
MAPK1	OV-7	4.860963	5.579241
MAPK1	SK-OV-3	6.0054	6.048105
MAPK1	IGROV-1	4.36807	5.465322
MAPK1	TOV-112D	5.159064	5.223423
MAPK1	OVCAR-3	4.474436	5.249825
MAPK1	OV-90	2.568032	5.380591
MAPK1	OVCAR-4	3.028569	5.024142
MAPK1	OAW42	3.529821	5.308885
MAPK1	OVCAR-5	3.037382	4.785551
MAPK1	OVISE	3.862947	4.171527
MAPK1	OV-17R	3.97728	3.28244
MAPK1	UWB1.289	3.514753	3.894333
MAPK1	OC-314	4.066089	4.768714
MAPK1	JHOS-2	4.520422	4.883132
MAPK1	JHOS-4	5.495056	5.414136
MAPK1	CAOV-4	4.58376	4.511595
MAPK1	FU-OV-1	5.018812	4.705425
MAPK1	PEO1	2.70044	3.496974
SMARCB1	TOV-21G	4.543496	6.529977
SMARCB1	OVTOKO	3.739848	5.899901
SMARCB1	OVK18	4.987321	7.338068
SMARCB1	OV-56	4.361768	5.441616
SMARCB1	OVKATE	3.140779	4.992315
SMARCB1	CAOV-3	4.654779	7.850249
SMARCB1	OVCAR-8	5.051807	6.721373
SMARCB1	OAW28	6.127221	6.754487
SMARCB1	SW626	4.350497	5.824768
SMARCB1	OV-7	4.860963	6.635464
SMARCB1	SK-OV-3	6.0054	7.107897
SMARCB1	IGROV-1	4.36807	6.734032
SMARCB1	TOV-112D	5.159064	6.940049
SMARCB1	OVCAR-3	4.474436	6.776499
SMARCB1	OV-90	2.568032	6.913009

SMARCB1	OVCAR-4	3.028569	6.038261
SMARCB1	OAW42	3.529821	6.819029
SMARCB1	OVCAR-5	3.037382	5.776894
SMARCB1	OVISE	3.862947	5.642413
SMARCB1	OV-17R	3.97728	4.523562
SMARCB1	UWB1.289	3.514753	5.488322
SMARCB1	OC-314	4.066089	5.791814
SMARCB1	JHOS-2	4.520422	5.626439
SMARCB1	JHOS-4	5.495056	6.068456
SMARCB1	CAOV-4	4.58376	6.406843
SMARCB1	FU-OV-1	5.018812	5.958147
SMARCB1	PEO1	2.70044	4.898692
ACACA	OV-7	4.860963	5.297191
ACACA	OVCAR-4	3.028569	4.979568
ACACA	TOV-21G	4.543496	4.691534
ACACA	TOV-112D	5.159064	6.275938
ACACA	JHOS-4	5.495056	4.654206
ACACA	OC-314	4.066089	5.494096
ACACA	OVK18	4.987321	6.122466
ACACA	OV-56	4.361768	4.507795
ACACA	IGROV-1	4.36807	5.021035
ACACA	OVKATE	3.140779	4.08151
ACACA	OV-17R	3.97728	4.060047
ACACA	OVISE	3.862947	4.710393
ACACA	UWB1.289	3.514753	4.570463
ACACA	OAW42	3.529821	4.999549
ACACA	OVCAR-5	3.037382	5.435962
ACACA	OVCAR-3	4.474436	4.28762
ACACA	OVTOKO	3.739848	3.485427
ACACA	PEO1	2.70044	4.145677
ACACA	CAOV-4	4.58376	4.908333
ACACA	SK-OV-3	6.0054	5.511278
ACACA	OVCAR-8	5.051807	4.920293
ACACA	OAW28	6.127221	6.465158
ACACA	JHOS-2	4.520422	4.239551
ACACA	SW626	4.350497	4.778734
ACACA	CAOV-3	4.654779	4.32337
ACACA	OV-90	2.568032	4.78136
ACACA	FU-OV-1	5.018812	4.657068
MTA2	JHOS-2	4.520422	5.606442
MTA2	CAOV-3	4.654779	6.431957
MTA2	OVISE	3.862947	4.497612
MTA2	SW626	4.350497	5.813268
MTA2	OVCAR-4	3.028569	5.718636

MTA2	IGROV-1	4.36807	6.771357
MTA2	OVCAR-5	3.037382	6.134837
MTA2	OV-17R	3.97728	5.892391
MTA2	OVTOKO	3.739848	5.301588
MTA2	TOV-112D	5.159064	6.418359
MTA2	OVK18	4.987321	7.247263
MTA2	SK-OV-3	6.0054	6.317232
MTA2	UWB1.289	3.514753	5.729009
MTA2	OVKATE	3.140779	5.366322
MTA2	OVCAR-8	5.051807	6.409221
MTA2	TOV-21G	4.543496	6.058966
MTA2	OV-56	4.361768	5.411765
MTA2	OAW28	6.127221	6.713421
MTA2	PEO1	2.70044	5.397461
MTA2	OAW42	3.529821	5.753551
MTA2	OV-90	2.568032	6.394377
MTA2	FU-OV-1	5.018812	5.696828
MTA2	OV-7	4.860963	6.227472
MTA2	JHOS-4	5.495056	6.527946
MTA2	OVCAR-3	4.474436	6.598871
MTA2	OC-314	4.066089	5.81839
MTA2	CAOV-4	4.58376	5.272023
YRDC	TOV-21G	4.543496	3.964399
YRDC	OVTOKO	3.739848	3.536053
YRDC	OVK18	4.987321	4.78398
YRDC	OV-56	4.361768	4.210233
YRDC	OVKATE	3.140779	3.647315
YRDC	CAOV-3	4.654779	5.154616
YRDC	OVCAR-8	5.051807	4.161081
YRDC	OAW28	6.127221	4.280956
YRDC	SW626	4.350497	4.275752
YRDC	OV-7	4.860963	4.408712
YRDC	SK-OV-3	6.0054	3.9855
YRDC	IGROV-1	4.36807	3.423578
YRDC	TOV-112D	5.159064	4.011675
YRDC	OVCAR-3	4.474436	4.365273
YRDC	OV-90	2.568032	3.979111
YRDC	OVCAR-4	3.028569	3.868884
YRDC	OAW42	3.529821	4.331992
YRDC	OVCAR-5	3.037382	3.377124
YRDC	OVISE	3.862947	3.452859
YRDC	OV-17R	3.97728	4.541639
YRDC	UWB1.289	3.514753	3.937344
YRDC	OC-314	4.066089	3.582556

YRDC	JHOS-2	4.520422	3.889474
YRDC	JHOS-4	5.495056	4.466627
YRDC	CAOV-4	4.58376	4.072106
YRDC	FU-OV-1	5.018812	4.045268
YRDC	PEO1	2.70044	3.848998
INTS6	JHOS-2	4.520422	4.280956
INTS6	CAOV-3	4.654779	3.528571
INTS6	OVISE	3.862947	2.568032
INTS6	SW626	4.350497	3.588565
INTS6	OVCAR-4	3.028569	2.996389
INTS6	IGROV-1	4.36807	4.096768
INTS6	OVCAR-5	3.037382	3.890447
INTS6	OV-17R	3.97728	2.91265
INTS6	OVTOKO	3.739848	2.526069
INTS6	TOV-112D	5.159064	3.554589
INTS6	OVK18	4.987321	3.91934
INTS6	SK-OV-3	6.0054	3.89724
INTS6	UWB1.289	3.514753	4.404631
INTS6	OVKATE	3.140779	3.972693
INTS6	OVCAR-8	5.051807	4.783457
INTS6	TOV-21G	4.543496	3.64501
INTS6	OV-56	4.361768	5.552131
INTS6	OAW28	6.127221	4.19456
INTS6	PEO1	2.70044	2.748461
INTS6	OAW42	3.529821	3.017922
INTS6	OV-90	2.568032	3.288359
INTS6	FU-OV-1	5.018812	3.258519
INTS6	OV-7	4.860963	4.352617
INTS6	JHOS-4	5.495056	4.745237
INTS6	OVCAR-3	4.474436	3.772941
INTS6	OC-314	4.066089	4.279471
INTS6	CAOV-4	4.58376	3.078951
USP9X	JHOS-2	4.520422	3.419539
USP9X	CAOV-3	4.654779	4.678635
USP9X	OVISE	3.862947	4.328406
USP9X	SW626	4.350497	4.478972
USP9X	OVCAR-4	3.028569	4.059182
USP9X	IGROV-1	4.36807	4.818646
USP9X	OVCAR-5	3.037382	3.619413
USP9X	OV-17R	3.97728	3.173127
USP9X	OVTOKO	3.739848	3.389567
USP9X	TOV-112D	5.159064	4.948134
USP9X	OVK18	4.987321	4.089159
USP9X	SK-OV-3	6.0054	5.040892

USP9X	UWB1.289	3.514753	2.786596
USP9X	OVKATE	3.140779	4.181898
USP9X	OVCAR-8	5.051807	4.303781
USP9X	TOV-21G	4.543496	4.783457
USP9X	OV-56	4.361768	5.759955
USP9X	OAW28	6.127221	5.078097
USP9X	PEO1	2.70044	3.221877
USP9X	OAW42	3.529821	5.144046
USP9X	OV-90	2.568032	4.672425
USP9X	FU-OV-1	5.018812	4.699885
USP9X	OV-7	4.860963	4.403268
USP9X	JHOS-4	5.495056	4.1152
USP9X	OVCAR-3	4.474436	4.361066
USP9X	OC-314	4.066089	5.280956
USP9X	CAOV-4	4.58376	4.040016
ILK	JHOS-2	4.520422	6.489286
ILK	CAOV-3	4.654779	5.564683
ILK	OVISE	3.862947	6.070604
ILK	SW626	4.350497	5.905447
ILK	OVCAR-4	3.028569	6.331813
ILK	IGROV-1	4.36807	6.195151
ILK	OVCAR-5	3.037382	6.87664
ILK	OV-17R	3.97728	4.925525
ILK	OVTOKO	3.739848	6.287805
ILK	TOV-112D	5.159064	5.470862
ILK	OVK18	4.987321	6.368594
ILK	SK-OV-3	6.0054	7.053872
ILK	UWB1.289	3.514753	5.46238
ILK	OVKATE	3.140779	5.66562
ILK	OVCAR-8	5.051807	6.59902
ILK	TOV-21G	4.543496	7.035734
ILK	OV-56	4.361768	6.61132
ILK	OAW28	6.127221	6.114992
ILK	PEO1	2.70044	4.761285
ILK	OAW42	3.529821	6.006298
ILK	OV-90	2.568032	5.486714
ILK	FU-OV-1	5.018812	6.930383
ILK	OV-7	4.860963	7.720689
ILK	JHOS-4	5.495056	5.89724
ILK	OVCAR-3	4.474436	6.129695
ILK	OC-314	4.066089	6.265662
ILK	CAOV-4	4.58376	5.598127
TUBD1	TOV-21G	4.543496	2.925999
TUBD1	OVTOKO	3.739848	3.119356

TUBD1	OVK18	4.987321	3.231125
TUBD1	OV-56	4.361768	3.344828
TUBD1	OVKATE	3.140779	3.70044
TUBD1	CAOV-3	4.654779	3.61471
TUBD1	OVCAR-8	5.051807	3.295723
TUBD1	OAW28	6.127221	3.763412
TUBD1	SW626	4.350497	1.823749
TUBD1	OV-7	4.860963	2.292782
TUBD1	SK-OV-3	6.0054	3.543496
TUBD1	IGROV-1	4.36807	3.554589
TUBD1	TOV-112D	5.159064	4.569856
TUBD1	OVCAR-3	4.474436	3.613532
TUBD1	OV-90	2.568032	3.502076
TUBD1	OVCAR-4	3.028569	2.503349
TUBD1	OAW42	3.529821	2.87578
TUBD1	OVCAR-5	3.037382	2.140779
TUBD1	OVISE	3.862947	3.549669
TUBD1	OV-17R	3.97728	3.377124
TUBD1	UWB1.289	3.514753	3.423578
TUBD1	OC-314	4.066089	3.02148
TUBD1	JHOS-2	4.520422	3.550901
TUBD1	JHOS-4	5.495056	3.171527
TUBD1	CAOV-4	4.58376	3.044394
TUBD1	FU-OV-1	5.018812	3.152183
TUBD1	PEO1	2.70044	1.95977
RAB6A	JHOS-2	4.520422	6.259272
RAB6A	CAOV-3	4.654779	6.502394
RAB6A	OVISE	3.862947	5.367371
RAB6A	SW626	4.350497	6.110614
RAB6A	OVCAR-4	3.028569	6.31379
RAB6A	IGROV-1	4.36807	5.802452
RAB6A	OVCAR-5	3.037382	5.491212
RAB6A	OV-17R	3.97728	5.032982
RAB6A	OVTOKO	3.739848	6.240314
RAB6A	TOV-112D	5.159064	5.164304
RAB6A	OVK18	4.987321	6.065012
RAB6A	SK-OV-3	6.0054	6.716305
RAB6A	UWB1.289	3.514753	6.05398
RAB6A	OVKATE	3.140779	7.138937
RAB6A	OVCAR-8	5.051807	6.460251
RAB6A	TOV-21G	4.543496	6.201046
RAB6A	OV-56	4.361768	6.62088
RAB6A	OAW28	6.127221	6.600359
RAB6A	PEO1	2.70044	6.053546

RAB6A	OAW42	3.529821	5.02148
RAB6A	OV-90	2.568032	4.921246
RAB6A	FU-OV-1	5.018812	6.419707
RAB6A	OV-7	4.860963	5.882888
RAB6A	JHOS-4	5.495056	6.963474
RAB6A	OVCAR-3	4.474436	5.807097
RAB6A	OC-314	4.066089	5.830103
RAB6A	CAOV-4	4.58376	5.14527
PPP2CA	JHOS-2	4.520422	5.819157
PPP2CA	CAOV-3	4.654779	6.243745
PPP2CA	OVISE	3.862947	6.119771
PPP2CA	SW626	4.350497	5.77926
PPP2CA	OVCAR-4	3.028569	6.633431
PPP2CA	IGROV-1	4.36807	6.199476
PPP2CA	OVCAR-5	3.037382	6.533875
PPP2CA	OV-17R	3.97728	5.567119
PPP2CA	OVTOKO	3.739848	5.39163
PPP2CA	TOV-112D	5.159064	5.971544
PPP2CA	OVK18	4.987321	7.25569
PPP2CA	SK-OV-3	6.0054	7.027132
PPP2CA	UWB1.289	3.514753	5.781884
PPP2CA	OVKATE	3.140779	7.019257
PPP2CA	OVCAR-8	5.051807	7.437294
PPP2CA	TOV-21G	4.543496	6.22593
PPP2CA	OV-56	4.361768	6.207893
PPP2CA	OAW28	6.127221	6.375039
PPP2CA	PEO1	2.70044	4.915521
PPP2CA	OAW42	3.529821	6.334676
PPP2CA	OV-90	2.568032	6.085977
PPP2CA	FU-OV-1	5.018812	6.061128
PPP2CA	OV-7	4.860963	6.478325
PPP2CA	JHOS-4	5.495056	6.297742
PPP2CA	OVCAR-3	4.474436	6.369466
PPP2CA	OC-314	4.066089	5.726831
PPP2CA	CAOV-4	4.58376	6.389567
PPP2R2A	TOV-21G	4.543496	5.120186
PPP2R2A	OVTOKO	3.739848	4.916954
PPP2R2A	OVK18	4.987321	4.962549
PPP2R2A	OV-56	4.361768	5.768184
PPP2R2A	OVKATE	3.140779	4.896756
PPP2R2A	CAOV-3	4.654779	4.720278
PPP2R2A	OVCAR-8	5.051807	5.617945
PPP2R2A	OAW28	6.127221	4.64501
PPP2R2A	SW626	4.350497	5.819668

PPP2R2A	OV-7	4.860963	4.608809
PPP2R2A	SK-OV-3	6.0054	5.67101
PPP2R2A	IGROV-1	4.36807	5.878235
PPP2R2A	TOV-112D	5.159064	5.614415
PPP2R2A	OVCAR-3	4.474436	6.525599
PPP2R2A	OV-90	2.568032	4.321207
PPP2R2A	OVCAR-4	3.028569	5.037821
PPP2R2A	OAW42	3.529821	5.170326
PPP2R2A	OVCAR-5	3.037382	4.111031
PPP2R2A	OVISE	3.862947	3.866908
PPP2R2A	OV-17R	3.97728	5.259649
PPP2R2A	UWB1.289	3.514753	4.02148
PPP2R2A	OC-314	4.066089	5.696272
PPP2R2A	JHOS-2	4.520422	4.691534
PPP2R2A	JHOS-4	5.495056	4.297925
PPP2R2A	CAOV-4	4.58376	4.982765
PPP2R2A	FU-OV-1	5.018812	5.322289
PPP2R2A	PEO1	2.70044	4.106013
